# Effectiveness of a Quality Improvement Intervention on Reperfusion Treatment for Patients With Acute Ischemic Stroke: A Stepped-Wedge Cluster Randomized Clinical Trial

**DOI:** 10.1001/jamanetworkopen.2023.16465

**Published:** 2023-06-02

**Authors:** Chun-Juan Wang, Hong-Qiu Gu, Li-Xia Zong, Xin-Miao Zhang, Qi Zhou, Yong Jiang, Hao Li, Xia Meng, Xin Yang, Meng Wang, Xiao-Chuan Huo, Run-Qi Wangqin, Yu-Zhang Bei, Xiu-Hui Qi, Xiao-Yun Liu, Shi-Qiang Hu, Zhi-Min Wang, Xing-Quan Zhao, Yi-Long Wang, Li-Ping Liu, Xu-Dong Ma, Louise Morgan, Ying Xian, Lee H. Schwamm, Yong-Jun Wang, Zi-Xiao Li

**Affiliations:** 1 China National Clinical Research Center for Neurological Diseases, Beijing Tiantan Hospital, Beijing, China; 2 Vascular Neurology, Department of Neurology, Beijing Tiantan Hospital, Capital Medical University, Beijing, China; 3 National Center for Healthcare Quality Management in Neurological Diseases, Beijing Tiantan Hospital, Capital Medical University, Beijing, China; 4 Research Unit of Artificial Intelligence in Cerebrovascular Disease, Chinese Academy of Medical Sciences, Beijing, China; 5 Department of Interventional Neuroradiology, Beijing Tiantan Hospital, Capital Medical University, Fengtai District, Beijing, China; 6 Department of Neurology, Duke University Medical Center, Durham, North Carolina; 7 Department of Neurology, Liuyang Jili Hospital, Hunan, China; 8 Department of Neurology, Jilin Electric Power Hospital, Jilin, China; 9 Department of Neurology, the Second Hospital of Hebei Medical University, Hebei, China; 10 Department of Neurology, Zhengzhou Zhongkang Hospital, Henan, China; 11 Department of Neurology, Taizhou First People's Hospital, Zhejiang, China; 12 Neuro-intensive Care Unit, Department of Neurology, Beijing Tiantan Hospital, Capital Medical University, Beijing, China; 13 Bureau of Medical Administration of National Health Commission, Beijing, China; 14 International Quality Improvement Department, American Heart Association, Dallas, Texas; 15 Department of Neurology, The University of Texas Southwestern Medical Center, Dallas; 16 Department of Neurology, Massachusetts General Hospital, Harvard Medical School, Boston; 17 Clinical Center for Precision Medicine in Stroke, Capital Medical University, Beijing, China; 18 Chinese Institute for Brain Research, Beijing, China

## Abstract

**Importance:**

Reperfusion therapy is the most effective treatment for acute ischemic stroke but remains underused in China.

**Objective:**

To evaluate the effect of a problem-oriented, culturally adapted, targeted quality improvement intervention on reperfusion therapy for patients with acute ischemic stroke in China.

**Design, Setting, and Participants:**

In this stepped-wedge cluster randomized clinical trial, patients from 16 secondary and 33 tertiary hospitals in China with acute ischemic stroke within 6 hours of symptom onset were consecutively recruited between July 1, 2018, and June 30, 2020.

**Interventions:**

Hospitals were randomly assigned to 1 of 3 sequences to receive the targeted quality improvement intervention (n = 5689), in which workflow reconstruction was promoted to reduce in-hospital reperfusion treatment delays, or usual care (n = 6443), in which conventional stroke care was left to the discretion of the stroke team.

**Main Outcomes and Measures:**

The primary outcome was the reperfusion therapy rate, a composite outcome of intravenous recombinant tissue plasminogen activator (IV rtPA) or endovascular thrombectomy (EVT) for eligible patients who arrived within 3.5 or 4.5 hours of symptom onset. Secondary outcomes were the IV rtPA administration rate among eligible patients who arrived within 3.5 hours of symptom onset, the EVT rate among eligible participants who arrived within 4.5 hours of symptom onset, the proportion of patients with door-to-needle time within 60 minutes, the proportion of patients with door-to-puncture time within 90 minutes, in-hospital mortality, and 3-month disability as measured by a modified Rankin Scale score greater than 2.

**Results:**

All 12 132 eligible patients (mean [SD] age, 66 [12.1] years; 7759 male [64.0%]) completed the trial. The reperfusion rate was 53.5% (3046 of 5689) for the eligible patients in the intervention period and 43.9% (2830 of 6443) in the control period. No significant improvement in primary outcomes was found for the intervention after adjusting for cluster, period, and imbalanced baseline covariates (adjusted risk difference [ARD], 5.5%; 95% CI, −8.0% to 19.0%; adjusted odds ratio [AOR], 1.26; 95% CI, 0.72-2.21) or for the secondary outcomes. However, significant improvements were found in secondary hospitals for reperfusion therapy (1081 of 1870 patients [57.8%] vs 945 of 2022 patients [42.9%]; ARD, 19.0%; 95% CI, 6.4%-31.6%; AOR, 2.24; 95% CI, 1.29-3.88), IV rtPA administration (1062 of 1826 patients [58.2%] vs 916 of 2170 patients [42.2%]; ARD, 20.3%; 95% CI, 7.4%-33.1%; AOR, 2.37; 95% CI, 1.34-4.19), and EVT (51 of 231 patients [22.1%] vs 37 of 259 patients [14.3%]; ARD, 13.6%; 95% CI, 1.0%-26.3%; AOR, 3.03; 95% CI, 1.11-8.25) in subgroup analyses.

**Conclusions and Relevance:**

In this stepped-wedge cluster randomized clinical trial of patients with acute ischemic stroke in China, the use of a targeted quality improvement intervention compared with usual care did not improve the reperfusion therapy rate. However, the intervention may be effective in secondary hospitals.

**Trial Registration:**

ClinicalTrials.gov Identifier: NCT03578107

## Key Points

**Question:**

What is the effect of a problem-oriented, culturally adapted, targeted quality improvement intervention on reperfusion therapy, including intravenous thrombolysis or endovascular thrombectomy, for acute ischemic stroke in China?

**Findings:**

In this stepped-wedge cluster randomized clinical trial of 12 132 patients with acute ischemic stroke who were enrolled within 3.5 or 4.5 hours of onset from 16 secondary and 33 tertiary hospitals, the rate of reperfusion therapy for eligible patients was 53.5% in the intervention period and 43.9% in the control period; the improvement was not statistically significant after adjusting for cluster and period, but it was statistically significant when limited to the setting of secondary hospitals.

**Meaning:**

Among patients with acute ischemic stroke in China, a targeted quality improvement intervention did not improve the reperfusion therapy rate compared with usual care but may be effective in secondary hospitals, which may provide a direction for better resource allocation.

## Teaser

This stepped-wedge cluster randomized clinical trial examines the effectiveness of a problem-oriented, culturally adapted, targeted quality improvement intervention to improve reperfusion therapy for acute ischemic stroke among patients in China.

## Introduction

Stroke remains the leading cause of long-term disability worldwide.^1^ As the most populous country in the world, China has the highest estimated risk of lifetime stroke, and the disability-adjusted life-years due to stroke increased by 36.7% from 1990 to 2019.^2,3^ Reperfusion therapy, including intravenous thrombolysis (IVT)^4,5,6^ with recombinant tissue plasminogen activator (rtPA) and endovascular thrombectomy (EVT),^7,8^ is the most effective treatment for improving functional outcomes of acute ischemic stroke.^9,10,11^ However, the rate of intravenous rtPA (IV rtPA) administration within 4.5 hours after stroke onset among eligible patients was only 22.9%, and the rate of EVT was only 0.8% as reported by the Chinese Stroke Center Alliance data from 2015 to 2019,^12^ which was far below the IVT rate (>73.4%) between 2013 and 2015 and the EVT rate (>3.3%) in 2016 reported by Get With the Guidelines–Stroke in the US.^13,14^

Previous cluster randomized clinical trials of multifaceted quality improvement interventions to improve adherence to evidence-based performance measures in patients with acute ischemic stroke have shown an inconsistent effect on the rate of IVT, and the EVT rate was not evaluated.^15,16^ Other trials that specifically aimed to improve thrombolysis by comprehensive intervention have revealed inconsistent conclusions as well.^17,18,19,20^ The low rate of IVT and EVT was presumably caused by the unequal distribution of high-quality health resources,^21,22^ the unorganized stroke care in stroke centers that have just begun to be constructed in China,^23,24^ the eroded patient-physician trust encountered during structural and social transformations,^25^ and the inadequate medical reimbursement in transitioning health systems in China.^26^ In addition, the Target: Stroke Initiative, launched by the American Heart Association/American Stroke Association (AHA/ASA), markedly improved the workflow of stroke care and demonstrated the effectiveness of key best practice strategies for reperfusion therapy in stroke centers.^27,28,29^ However, whether these strategies would be effective in a different health care setting in China remains unknown. Therefore, we developed a problem-oriented, culturally adapted, targeted quality improvement intervention with the support of the AHA/ASA to improve the reperfusion rate for patients with acute ischemic stroke in China.^30^ To examine whether reperfusion therapy for patients with acute ischemic stroke could be improved with a problem-oriented, culturally adapted, targeted quality improvement intervention, we performed a pragmatic, stepped-wedge cluster randomized clinical trial for sequential implementation across hospitals in China.

## Methods

The trial protocol was approved by the central institutional review board at Beijing Tiantan Hospital, Capital Medical University. Written informed consent was obtained from patients or their proxies before enrollment. The study was conducted according to the study protocol (Supplement 1) and analyzed according to the statistical analysis plan (Supplement 1). This stepped-wedge cluster randomized clinical trial was reported according to the Consolidated Standards of Reporting Trials (CONSORT) reporting guideline.^31^

### Trial Design and Oversight

The detailed study design has been previously reported.^30^ Briefly, the Improve Acute Reperfusion Treatment Quality for Stroke in China (IMPROVE Stroke Care in China) trial was a stepped-wedge, pragmatic cluster randomized clinical trial in which 51 hospitals (clusters) in China were randomly assigned to receive a problem-oriented, culturally adapted, and targeted quality improvement intervention for 1 of 3 predefined, 6-month periods during an 18-month period between January 1, 2019, and June 30, 2020, after a 6-month period of usual care.

### Hospitals and Study Participants

We planned to recruit 51 hospitals in mainland China from the Chinese Stroke Center Alliance hospitals located in 29 provinces, autonomous regions, and municipalities. To ensure the diversity and representativeness of the included hospitals, hospitals were enrolled considering their economic-geographic regions (Eastern, Central, and Western) and hospital level (secondary or tertiary). To be eligible, hospitals were required to be secondary or tertiary public hospitals with emergency department and neurologic wards, have a 24/7 on-call stroke team, have the capacity for IVT or EVT, have administered IVT or EVT to at least 10 patients during the whole year before enrollment, and have at least 5 patients admitted within 6 hours after stroke onset per month. All hospitals had to want to improve the workflow of treating patients with acute ischemic stroke and have good collaboration among the multidisciplinary team. Hospitals that participated in other research projects of reperfusion quality improvement in patients with acute ischemic stroke were all excluded after a questionnaire survey.

At participating hospitals, patients were consecutively enrolled if they were older than 18 years; presented with symptoms of acute ischemic stroke, which was confirmed by computed tomography (CT) or magnetic resonance imaging (MRI); and arrived at the hospital within 6 hours of symptom onset.^30^ Patients diagnosed with other cerebrovascular diseases, such as transient ischemic attack (TIA), hemorrhagic stroke, cerebral venous sinus thrombosis, or noncerebrovascular diseases, were excluded. All patients were scheduled to be followed up for 90 days to obtain information on the modified Rankin Scale (mRS) score (range, 0 [no symptoms] to 6 [death]) or death through telephone interviews by a blinded centralized follow-up team who was trained by the research team.

### Randomization

The cluster randomization was performed at the hospital level centrally using a computer-generated random number sequence. The other members of the study team and the selected sites were informed that they would cross over to the intervention period 1 month before each of the predefined steps to maintain allocation concealment while aiding in training logistics. A total of 17 hospitals were randomly assigned to 1 of the 3 sequences (cohorts) considering hospital location and level to minimize potential imbalance between the intervention and control periods as much as possible. Of the 51 participating hospitals (17 secondary and 34 tertiary hospitals), 2 (1 secondary hospital and 1 tertiary hospital) withdrew from this trial because they were unable to recruit eligible patients, and 1 hospital discontinued the study before the intervention and was replaced by a newly enrolled hospital with the same hospital level.

### Intervention and Training

A problem-oriented, culturally adapted, targeted quality improvement intervention, STEP (strategies, toolkit, exploration, and paradigm), was developed based on the theories of implementation science and the consolidated framework of implementation research^32^ to promote workflow reconstruction in stroke centers and to shorten in-hospital delays of reperfusion treatment for patients with acute ischemic stroke (eFigure 1 in Supplement 2). More detailed descriptions of the STEP intervention are reported in the eMethods in Supplement 2 and the protocol (Supplement 1).^30^

### Outcome Measures

The primary outcome was reperfusion therapy rate, defined as a composite outcome of IVT for eligible patients who arrived within 3.5 hours of symptom onset or EVT for eligible patients who arrived within 4.5 hours of symptom onset. The secondary outcomes included the rate of IV rtPA administration among eligible patients who arrived within 3.5 hours of symptom onset; the rate of EVT among eligible patients who arrived within 4.5 hours of symptom onset; the proportion of patients with a door-to-needle time (DNT) within 60 minutes among participants who received IV rtPA; the proportion of patients with a door-to-puncture time (DPT) within 90 minutes among participants who received EVT; in-hospital mortality; and 3-month disability as measured by an mRS score greater than 2.

### Sample Size

Assuming 31 patients per period (6 months) per hospital, 51 hospitals, a 0.03 intraclass correlation coefficient, and a 19% reperfusion treatment rate at baseline, we expected a total of 6324 patients would provide 90% statistical power for detecting a 30% improvement in the primary outcomes (from 19% to 25%) at a 2-sided α level of 0.05. The 19% rate was based on the Chinese Stroke Center Alliance data up to March 2018.^12^ However, because of the increasing enrollment volumes of participating hospitals in clinical practice and the continuous recruitment design, the final enrollment was much larger than the initial targeted sample size, which may provide abundant power for this analysis.

### Statistical Analysis

All results were reported using an intention-to-treat analysis. Baseline characteristics are summarized as the mean (SD) or median (IQR) for continuous variables and as numbers (percentages) for categorical variables. Baseline characteristics between the intervention and control participants were compared using crude differences and 95% CIs.

We first performed the analyses for the primary analysis using mixed-effects logistic regression models with a random effect for the cluster (hospital) and a fixed-time effect for every 6-month period. However, the models did not converge. Therefore, we modeled the outcomes using generalized estimating equation models to account for the clustering effect within hospitals, with a fixed-time effect for the period. To check the robustness of our primary analysis, we also modeled the outcomes using generalized estimating equation models with additional adjustment for baseline imbalanced covariates (covariate-adjusted analysis), including medical history of stroke or TIA, medication history of antiplatelet agent and statin, and hospital location. In all these analyses, we reported the absolute effect measure risk difference (RD) and 95% CI estimated from a binomial regression model with the identity link function, in addition to the relative effect measure odds ratio (OR) and 95% CI.^33^

A total of 1423 of 11 821 participants discharged alive (12.0%) had missing data for mRS scores at 90 days; therefore, we first reported the results based on the complete data and then used multiple imputations by fully conditional specification to generate 5 complete data sets. The imputation models included all baseline covariates listed in Table 1. The analyses were then performed for each data set separately, and the results were pooled according to the Rubin rules.^34^

**Table 1.** Baseline Patient and Hospital Characteristics of the Improve Acute Reperfusion Treatment Quality for Stroke in China (IMPROVE Stroke Care in China) Trial^a^

Characteristic	Intervention (n = 5689 [46.9%])	Control (n = 6443 [53.1%])	Difference (95% CI)
**Patient characteristics**			
Demographic characteristics			
Age, mean (SD), y	66.4 (12.1)	65.9 (12.0)	0.5 (0.1 to 0.9)
Sex			
Male	3655 (64.2)	4104 (63.7)	0.5 (−1.2 to 2.3)
Female	2034 (35.8)	2339 (36.3)	−0.5 (−2.3 to 1.2)
BMI, mean (SD)	23.6 (3.5)	23.9 (4.0)	−0.3 (−0.4 to −0.2)
NIHSS score at admission, median (IQR)	5.0 (2.0 to 10.0)	5.0 (2.0 to 10.0)	0.0 (0.0 to 0.0)
Current smoking	1570 (27.6)	1668 (25.9)	1.7 (0.1 to 3.3)
Drinking	1386 (24.4)	1607 (24.9)	−0.5 (−2.1 to 1.0)
Medical history			
Stroke or TIA	1358 (23.9)	2087 (32.4)	−8.5 (−10.1 to −6.9)
Hypertension	3435 (60.4)	4022 (62.4)	−2.0 (−3.8 to −0.3)
Diabetes	1092 (19.2)	1309 (20.3)	−1.1 (−2.5 to −0.3)
Dyslipidemia	301 (5.3)	424 (6.6)	−1.3 (−2.1 to −0.5)
Myocardial infarction	117 (2.1)	125 (1.9)	0.2 (−0.4 to 0.6)
Atrial fibrillation	579 (10.2)	528 (8.2)	2.0 (1.0 to 3.0)
Peripheral vascular disease	52 (0.9)	113 (1.8)	−0.9 (−1.2 to −0.4)
Medication history			
Antiplatelet agent	904 (15.9)	1346 (20.9)	−5.0 (−6.4 to −3.6)
Anticoagulant drugs	182 (3.2)	370 (5.7)	−2.5 (−3.3 to −1.8)
Antihypertensive drugs	2501 (44.0)	2876 (44.6)	−0.6 (−2.4 to 1.1)
Antidiabetics	853 (15.0)	1051 (16.3)	−1.3 (−2.6 to 0.0)
Statins	749 (13.2)	1212 (18.8)	−5.6 (−6.9 to −4.3)
Onset-to-door time, median (IQR), min	95.0 (55.0 to 144.0)	94.0 (54.0 to 146.0)	−2.0 (−4.0 to 0.0)
**Hospital characteristics**			
Hospital level			
Tertiary	3819 (67.1)	4241 (65.8)	1.3 (−0.4 to 3.0)
Secondary	1870 (32.9)	2202 (34.2)	−1.3 (−3.0 to 0.4)
Hospital location			
Eastern	2385 (41.9)	3189 (49.5)	−7.6 (−9.3 to −5.8)
Central	1857 (32.6)	1807 (28.0)	4.6 (3.0 to 6.2)
Western	1447 (25.4)	1447 (22.5)	2.9 (1.5 to 4.5)

Abbreviations: BMI, body mass index (calculated as weight in kilograms divided by height in meters squared); NIHSS, National Institutes of Health Stroke Scale; TIA, transient ischemic attack.

^a^ Data are presented as number (percentage) unless otherwise indicated.

To check the heterogeneity between subgroups, we performed post hoc subgroup analyses by age (<65 or ≥65 years), sex, and hospital level (secondary or tertiary) in the abovementioned primary and covariable-adjusted analysis. *P* values were calculated from generalized estimating equation models. A 2-sided *P* < .05 was considered statistically significant. We conducted all analyses using SAS software, version 9.4 (SAS Institute Inc).

## Results

**Figure 1.**
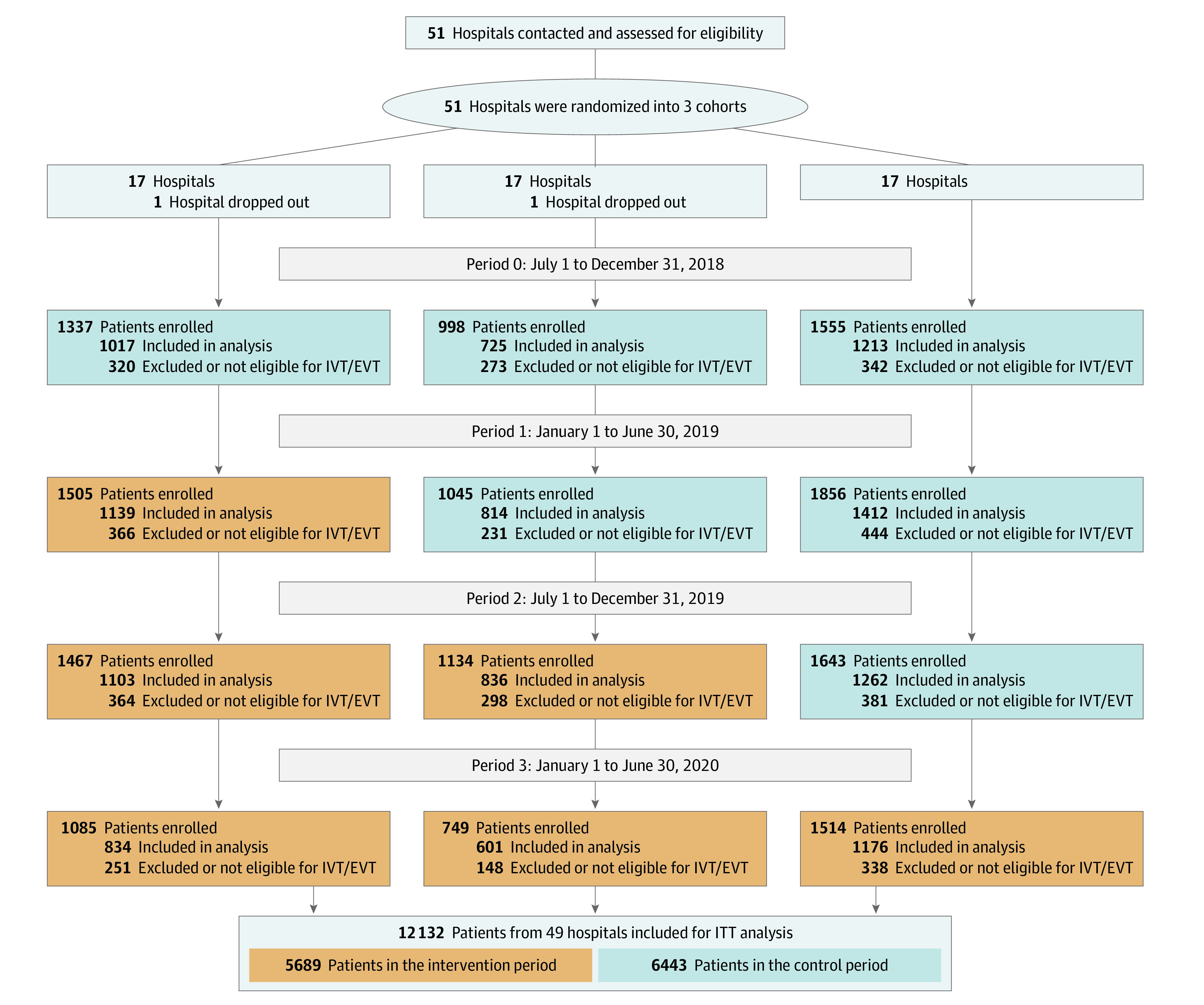
Flow of Hospitals and Participants Through the Improve Acute Reperfusion Treatment Quality for Stroke in China (IMPROVE Stroke Care in China) EVT indicates endovascular thrombectomy; ITT, Intention to treat; and IVT, intravenous thrombolysis.

A total of 12 132 participants (5689 [46.9%] in the intervention period and 6443 [53.1%] in the control period) were included in this analysis. The mean (SD) age of the participants was 66.1 (12.1) years; 7759 (64.0% were men and 4373 (36.0%) were women. Details on patient enrollment and identification are shown in Figure 1. Baseline characteristics between the included and excluded participants were largely comparable except that the former group had more patients recruited from hospitals located in Eastern China and fewer patients from Central China (eTable 1 in Supplement 2).

### Patient Characteristics

The median (IQR) National Institutes of Health Stroke Scale score (range, 0-42, with a higher score indicating greater stroke severity) at admission was 5 (2-10). A total of 7457 participants (61.5%) had a medical history of hypertension, 3445 (28.4%) had a history of stroke or TIA, and 2401 (19.8%) had diabetes. The baseline characteristics between the intervention and control periods were generally similar except for a lower proportion of patients with a medical history of stroke or TIA (difference, −8.5%; 95% CI, −10.1% to −6.9%), patients with prior use of antiplatelet agents (difference, −5.0%; 95% CI, −6.4% to −3.6%) and statins (difference, −5.6%; 95% CI, −6.9% to −4.3%), and patients recruited from hospitals located in Eastern China (difference, −7.6%; 95% CI, −9.3% to −5.8%) in the former group (Table 1).

### Primary and Secondary Outcomes

**Figure 2.**
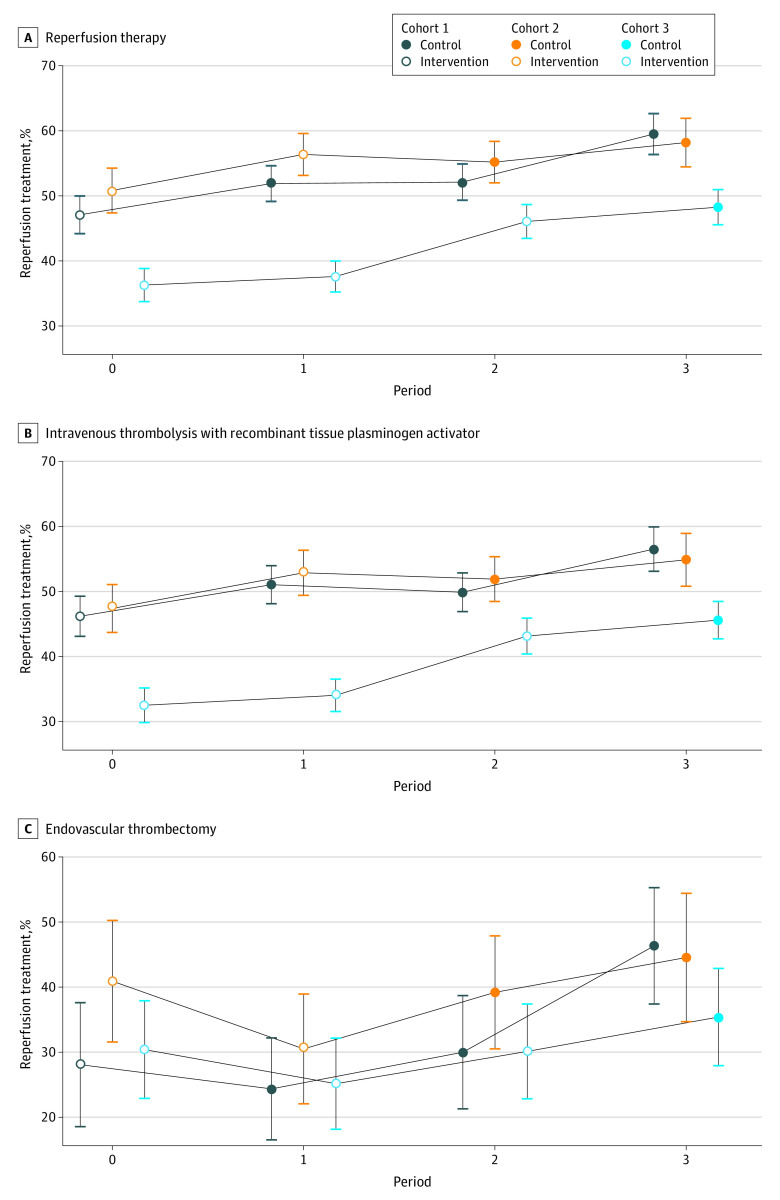
Unadjusted Temporal Trends in Reperfusion Therapy A, Reperfusion therapy among eligible patients who arrived within 3.5 or 4.5 hours of symptom onset. B, Intravenous thrombolysis among eligible patients who arrived within 3.5 hours of symptom onset. C, Endovascular thrombectomy among eligible patients who arrived within 4.5 hours of symptom onset. Whiskers indicate 95% CIs.

A total of 3046 of 5689 participants (53.5%) in the intervention period and 2830 of 6443 (43.9%) in the control period received reperfusion therapy. The temporal trend for reperfusion therapy is shown in Figure 2A. After adjustment for cluster and period, the implementation of the STEP intervention did not result in a significantly higher rate of reperfusion therapy (adjusted RD [ARD], 7.8%; 95% CI, −7.3% to 23.0%; adjusted OR [AOR], 1.37; 95% CI, 0.74-2.52) (Table 2). The results from the imbalanced baseline covariate-adjusted analysis were consistent with those from the primary analysis (ARD, 5.5%; 95% CI, −8.0% to 19.0%; AOR, 1.26; 95% CI, 0.72-2.21) (Table 2).

**Table 2.** Primary and Secondary Trial Outcomes

Outcomes	ICC	No. of cases/total No. (%)		Primary analysis^a^		Covariate-adjusted analysis^b^	
		Intervention	Control	ARD (95% CI)	AOR (95% CI)	ARD (95% CI)	AOR (95% CI)
**Primary outcome**							
Reperfusion therapy among patients who arrived within 3.5 or 4.5 h of symptom onset	0.28	3046/5689 (53.5)	2830/6443 (43.9)	7.8 (−7.3 to 23.0)	1.37 (0.74 to 2.52)	5.5 (−8.0 to 19.0)	1.26 (0.72 to 2.21)
**Secondary outcomes**							
IV rtPA among patients who arrived within 3.5 h of symptom onset	0.28	2830/5540 (51.1)	2612/6316 (41.4)	8.9 (−6.4 to 24.2)	1.43 (0.77 to 2.66)	6.7 (−7.0 to 20.5)	1.33 (0.75 to 2.36)
EVT among patients who arrived within 4.5 h of symptom onset	0.36	270/739 (36.5)	236/772 (30.6)	0.8 (−11.7 to 13.4)	1.05 (0.57 to 1.95)	0.4 (−10.1 to 11.0)	0.99 (0.57 to 1.71)
DNT within 60 min	0.22	2018/2830 (71.3)	1701/2612 (65.1)	7.5 (−6.0 to 21.0)	1.44 (0.76 to 2.75)	7.1 (−5.3 to 19.4)	1.39 (0.77 to 2.54)
DPT within 90 min	0.23	62/270 (23.0)	86/236 (36.4)	−25.6 (−40.6 to −10.7)	0.26 (0.12 to 0.57)	−18.4 (−29.4 to −7.3)	0.27 (0.14 to 0.52)
In-hospital death	0.14	146/5689 (2.6)	165/6443 (2.6)	−0.2 (−1.3 to 0.9)	0.92 (0.58 to 1.45)	−0.3 (−1.2 to 0.6)	0.93 (0.61 to 1.41)
Disability at 90 d^c^	0.02	1215/4687 (25.9)	1439/5711 (25.2)	−1.3 (−5.4 to 2.9)	0.94 (0.75 to 1.16)	−0.9 (−5.1 to 3.2)	0.96 (0.77 to 1.20)

Abbreviations: AOR, adjusted odds ratio; ARD, adjusted risk difference; DNT, door-to-needle time; DPT, door-to-puncture time; EVT, endovascular thrombectomy; ICC, intracluster correlation; IV rtPA, intravenous recombinant tissue plasminogen activator.

^a^ Results were yielded from generalized estimating equation logistic regression models and adjusted for the hospital clustering effect and temporal trend (period).

^b^ Results were yielded from generalized estimating equation logistic regression models and adjusted for the hospital clustering effect, temporal trend (period), and imbalanced baseline covariates (medical history of stroke or transient ischemic attack, medication history of antiplatelet agent and statin, and hospital location).

^c^ A total of 1423 of 11 821 patients discharged alive (12.0%; 856 of 5543 [15.4%] in the intervention period vs 567 of 6278 [9.0%] in the control period) had missing modified Rankin Scale scores because of loss of follow-up.

We also assessed the components of reperfusion therapy. The rate of IV rtPA administration among eligible patients who arrived within 3.5 hours of symptom onset was 51.1% (2830 of 5540) in the intervention period compared with 41.4% (2612 of 6316) in the control period; the rate of EVT among eligible patients who arrived within 4.5 hours of symptom onset was 36.5% (270 of 739) in the intervention period compared with 30.6% (236 of 772) in the control period. Figure 2B and C shows the temporal trends for IV rtPA administration and EVT, respectively. The difference in the rate of IV rtPA administration (ARD, 8.9%; 95% CI, −6.4% to 24.2%; AOR, 1.43; 95% CI, 0.77-2.66) or EVT (ARD, 0.8%; 95% CI, −11.7% to 13.4%; AOR, 1.05; 95% CI, 0.57-1.95) between the intervention and control periods was not significant after adjusting for the cluster and period. The imbalanced baseline covariate-adjusted analyses show similar results (Table 2). Improvements in other secondary outcomes were not statistically significant, including a DNT within 60 minutes, a DPT within 90 minutes, in-hospital death, and disability at 90 days.

Baseline characteristics between patients with and without mRS scores at 90 days were largely comparable (eTable 2 in Supplement 2). Therefore, we also reported sensitivity analysis from multiple imputed data for mRS scores at 90 days, which revealed comparable results with previous analyses (eTable 3 Supplement 2).

### Subgroup Analysis

**Figure 3.**
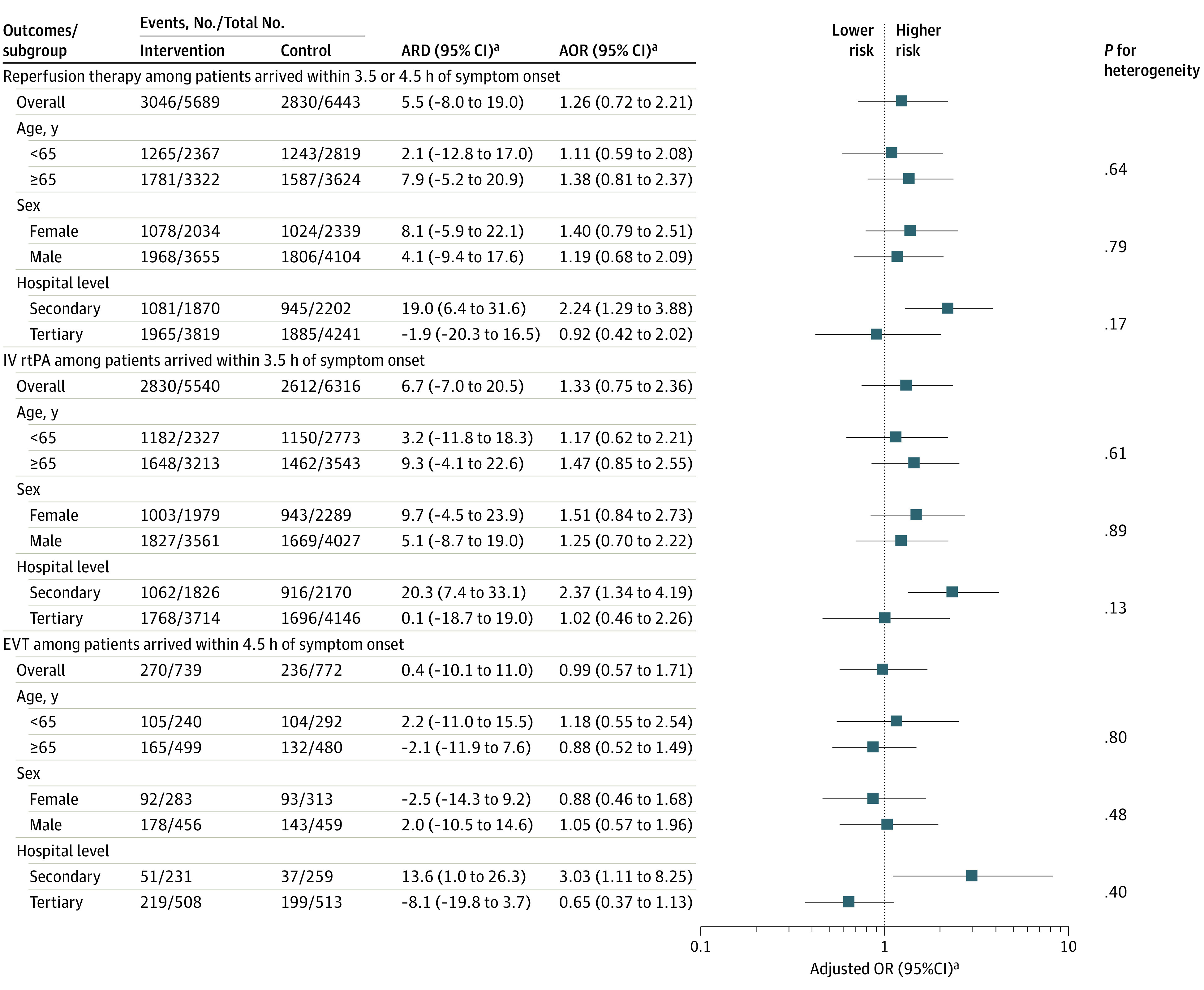
Subgroup Analysis for the Effect of Intervention on Reperfusion Therapy AOR indicates adjusted odds ratio; ARD, adjusted risk difference; EVT, endovascular thrombectomy; IV rtPA, intravenous recombinant tissue plasminogen activator; and OR, odds ratio. ^a^Results were yielded from generalized estimating equation logistic regression models and adjusting for a medical history of stroke or transient ischemic attack, medication history of antiplatelet agent and statin, and hospital location.

No significant statistical heterogeneity was found in the intervention effect by age, sex, or hospital level. However, we found that the intervention among secondary hospitals resulted in significantly higher rates of reperfusion therapy (1081 of 1870 patients [57.8%] vs 945 of 2202 patients [42.9%]; ARD, 19.0%; 95% CI, 6.4% to 31.6%; AOR, 2.24; 95% CI, 1.29-3.88), intravenous thrombolysis (1062 of 1826 [58.2%] vs 916 of 2170 patients [42.2%]; ARD, 20.3%; 95% CI, 7.4% to 33.1%; AOR, 2.37; 95% CI, 1.34-4.19), and EVT (51 of 231 patients [22.1%] vs 37 of 259 patients [14.3%]; ARD, 13.6%; 95% CI, 1.0%-26.3%; AOR, 3.03; 95% CI, 1.11-8.25) in the intervention period after adjusting for cluster, period, and imbalanced baseline covariates (Figure 3).

No significant heterogeneity was found in the intervention effect by subgroup for the other secondary outcomes, except that the intervention significantly reduced the rate of in-hospital death (0.8% vs 1.2%; ARD, −0.8%; 95% CI, −1.5% to −0.1%; AOR, 0.39; 95% CI, 0.18-0.83) and may have reduced the rate of disability at 90 days (14.8% vs 16.2%; ARD, −2.8%; 95% CI, −5.7% to 0.2%; AOR, 0.81; 95% CI, 0.63-1.02) among patients younger than 65 years but not among those 65 years or older (eTable 4 in Supplement 2).

## Discussion

In this stepped-wedge cluster randomized clinical trial, we found that the STEP targeted quality intervention did not increase the overall rate of reperfusion therapy, the rate of a DNT within 60 minutes, or the rate of a DPT within 90 minutes. However, it increased the rate of reperfusion therapy in secondary hospital settings. The results showed that the problem-oriented, culturally adapted, targeted quality intervention might be more effective in secondary hospital settings, which may provide a direction for better resource allocation. According to the latest statistics, there are more than 10 000 secondary hospitals in China,^35^ and more than 27 million patients with acute ischemic stroke are admitted to secondary hospitals yearly.^36^ Therefore, providing more resources to resource-constrained secondary hospitals to efficiently improve reperfusion therapy adoption would benefit most patients with acute ischemic stroke in China and have profound implications for the whole health care system and public health.

There are several potential reasons why the STEP targeted quality improvement intervention was not effective for the improvement of reperfusion therapy overall but may be effective for secondary hospitals. First, because the effect of reperfusion therapy was confirmed in 5 milestone trials in 2015,^7^ efforts for the adoption of reperfusion therapy have been advocated for by national and local health authorities and medical associations in China, which finally led to increasing the rate of reperfusion therapy. In 2021, increasing the rate of reperfusion therapy became 1 of the 10 National Health Quality and Safety Improvement Goals released by the National Health Commission of the People's Republic of China.^37^ Although the enrollment of our trial was completed before the start of the goals, under this circumstance, the temporal trend of the rate of reperfusion therapy has been increasing steadily, as data from the Chinese Stroke Center Alliance Nationwide registry^38^ illustrates (eFigure 2 in Supplement 2), which made it even harder for the STEP targeted quality improvement intervention to show the overall effect. Second, the admission of eligible patients in this study in the last 6 months was reduced because of the COVID-19 pandemic. During this time, most of the medical resources (especially staff) involved in this trial were redirected to the COVID-19 outbreak in China, which seriously influenced the implementation of strategies, the exploration of expert guidance, and the use of the Plan-Do-Study-Act paradigm.^39^ Therefore, the total effect size of the intervention may have been reduced as well. Third, the baseline reperfusion rate (43.9%) in our trial was much higher than estimated and higher than the rates in other hospitals, which may suggest another reason why the STEP intervention is not effective overall. Fourth, almost 2 times more hospitals and participants were enrolled in tertiary hospitals than in secondary hospitals. Because tertiary hospitals receive more research projects and physicians are more overworked than that in secondary hospitals,^40^ participating in research programs and obtaining training is not as incentivizing for physicians in tertiary hospitals compared with those in secondary hospitals; therefore, the implementation or adoption of the intervention may be compromised in tertiary hospitals. This issue could be another reason why the intervention was not effective in tertiary hospital settings but was effective in secondary hospital settings.

We noted that fewer patients had a DPT within 90 minutes in the intervention period than in the control period, which may be explained by the higher proportion of patients having a DPT within 90 to 150 minutes in the intervention period (eFigure 3 in Supplement 2). The intervention did not result in a lower rate of disability at 90 days. However, analysis of the effect of reperfusion therapy showed a lower risk of disability at 90 days after adjustment for imbalanced baseline covariates (ARD, −4.0%; 95% CI, −6.1% to −1.9; AOR, 0.80; 95% CI, 0.70-0.91) (eTables 5 and 6 in Supplement 2).

Several studies focusing on the improvement of thrombolysis have been launched. The TIPS (Thrombolysis Implementation in Pediatric Stroke) trial,^17^ the AVC II (Impact of a Training Program and Organization on the Management of Stroke in the Acute Phase II) trial,^19^ and the PASTA (Paediatric Arteriopathy Steroid Aspirin Project) trial^20^ aimed for the adoption of thrombolysis through comprehensive intervention and yielded inconsistent results. The ANGEL-ACT (Endovascular Treatment Key Technique and Emergency Work Flow Improvement of Acute Ischemic Stroke)^41^ and IMPACT (Improving Patient Access to Stroke Therapy Study) (NCT01870492) studies aim to identify barriers to reperfusion therapy through observation studies, whereas the MISSION (Multicomponent Intervention to Shorten Thrombolytic Door-to-Needle Time in Stroke Patients in China) trial^18^ and the Target: Stroke trial^27^ aim to shorten DNT, and the ANGEL-ACT II trial^41^ aims to shorten DPT through multimodal medical quality improvement by a cluster randomized, parallel controlled design. To the best of our knowledge, the IMPROVE Stroke Care trial is the first and most well-powered clinical trial to evaluate a problem-oriented, culturally adapted, targeted quality improvement intervention for increasing the adoption of reperfusion therapy among eligible patients with acute ischemic stroke in China.

### Strengths and Limitations

The IMPROVE Stroke Care trial has several strengths. First, the STEP intervention package was created based on potential causes of China’s low rate of reperfusion therapy, as well as incorporating some successful practices from other countries. Second, it was built on previous observational data and the organization of the National Registry Program Chinese Stroke Center Alliance,^12,38^ which provided a solid foundation for the design and implementation of the trial. Third, the study used a stepped-wedge, cluster-randomized, pragmatic trial design, allowing all hospitals to eventually receive the intervention, which would maximally benefit clinical practice. Fourth, the causal inference was notably improved with appropriate and strict statistical methods and elaborate sensitivity analysis.

This study also has several limitations. First, the rate of reperfusion therapy and the intraclass correlation coefficient are higher than those estimated in the sample size estimation stage. However, the final sample size was much larger than the planned sample size because of the increasing enrollment volumes of participating hospitals in clinical practice and the continuous recruitment design, which made the trial well powered to detect the current difference. Second, the COVID-19 pandemic slowed enrollment during the last period of the trial, which increased the imbalance between the intervention and control period and may also have introduced enrollment bias. However, the baseline characteristics were largely comparable. The COVID-19 pandemic led to difficulties in the implementation of the intervention, which may also have impaired the intervention effect. Third, the complex intervention included many components (eg, workflow reconstruction, toolkits, technical trainings, and workshops) that may not have been well received and fully implemented at all sites, especially at tertiary hospitals, or not implemented for a sufficient duration to take effect. On the basis of the survey after the intervention, a single call activation of the stroke team was implemented in only approximately 75% of hospitals (73% in tertiary vs 80% in secondary hospitals), stroke-specific order sets were used in only 77% (72% in tertiary vs 83% in secondary hospitals), and a weekly or biweekly improvement meeting was held in 85.4% (78% in tertiary vs 89% in secondary hospitals). Fourth, the effect of intervention was seen in secondary hospitals. However, this result was drawn from the post hoc subgroup analysis without prespecified sample size calculation or adjustment for multiple subgroup tests, and the interaction *P* value was greater than .10. Therefore, this result should be interpreted as an exploratory analysis.

## Conclusions

Among patients with acute ischemic stroke, the implication of a problem-oriented and culturally adapted, targeted quality improvement intervention compared with usual care at the hospital level did not result in a statistically significant improvement in the rate of reperfusion therapy. However, the intervention may be effective in resource-constrained secondary hospitals. Additional randomized clinical trials are needed to confirm the efficacy of the targeted quality improvement intervention among secondary hospitals, and further analyses are needed to understand the mechanism underlying the lack of efficacy in tertiary hospitals.

## Notes

Supplement 1.
Trial Protocol and Statistical Analysis Plan

Supplement 2.
eMethods.
**eTable 1.** Baseline Characteristics of the IMPROVE Stroke Care in China Patients and Hospitals Between Included and Excluded Patients **eTable 2.** Baseline Characteristics of the IMPROVE Stroke Care China Patients and Hospitals Among Patients With and Without Values of mRS at 90 Days **eTable 3.** Results of Disability at 90 Days From Multiple Imputations **eTable 4.** Subgroup Analysis for the Effect of Intervention on Reperfusion Treatment for Other Secondary Outcomes **eTable 5.** Baseline Characteristics of the IMPROVE Stroke Care in China Patients and Hospitals by Reperfusion **eTable 6.** Results of Disability at 90 Days From Raw Data **eFigure 1.** The Design of “STEP” Intervention **eFigure 2.** Reperfusion Therapy in the Chinese Center Association Alliance Hospitals Not Participating in the IMPROVE Stroke Care in China Trial **eFigure 3.** Distribution of Door-to-Puncture Time in the IMPROVE Stroke Care In China Trial

Supplement 3.
Nonauthor Collaborators

Supplement 4.
Data Sharing Statement
