# Supplementary material for: Effectiveness of a Quality Improvement Intervention on Reperfusion Treatment for Patients With Acute Ischemic Stroke: A Stepped-Wedge Cluster Randomized Clinical Trial
Source: JAMA Netw Open. 2023 Jun 2;6(6):e2316465. doi: 10.1001/jamanetworkopen.2023.16465 (PMC10238948; doi:10.1001/jamanetworkopen.2023.16465)
Supplement: Supplement 1. — Trial Protocol and Statistical Analysis Plan [file jamanetwopen-e2316465-s001.pdf]

IMPROVE ACUTE REPERFUSION TREATMENT QUALITY FOR STROKE: IMPROVE STROKE CARE IN CHINA

A STEPPED WEDGE CLUSTER RANDOMIZED TRIAL

Principal Investigator:
Prof. Yongjun Wang
Neurology Center, Beijing Tiantan Hospital, Capital Medical University
China National Clinical Research Center for Neurological Diseases
No. 119, the fourth southwest ring road, Fengtai District, Beijing, China, 100070

Study Coordinating Group and Central Address:
China National Clinical Research Center for Neurological Diseases
No. 119, the fourth southwest ring road, Fengtai District, Beijing, China, 100070

Date of Protocol: July 1, 2018

Sponsors: America Heart Association/America Stroke Association and China Stroke Association

Protocol Synopsis

**Title of the Study**	Improve Acute Reperfusion Treatment Quality for Stroke: Improve Stroke Care In China
**Short Title**	IMPROVE
**Study Sponsor**	China Stroke Association and America Heart Association / America Stroke Association
**Principal Investigator**	Prof. Yongjun Wang
**Central Coordinating Centre**	China National Clinical Research Center for Neurological Diseases
**Recruitment and Participating Clinical Sites**	Approximately 7644 participants and 51 Hospitals in China
**Phase/Regulatory Status**	Phase IV
**Study Goal**	This goal of this study is to investigate the effectiveness of a comprehensive quality improvement intervention on adherence to guideline-recommended reperfusion therapy for acute ischemic stroke (AIS) patients in China.
**Study Objectives**	To improve the rate of adherence to reperfusion treatment, including intravenous thrombolysis (IVT)/ endovascular thrombectomy (EVT) for eligible patients.
**Study Design**	The IMPROVE trial is designed as a **stepped wedge** cluster randomized trial within 51 hospitals. The participating hospitals (clusters) were randomized to three groups (cohorts) for different predefined steps to intervention implementation. Hospital location and grade will be matched during the randomization to minimize imbalances throughout the trial. A confirmation letter will be sent to all the hospitals in a particular cohort one month before the implementation of the intervention to ensure preparedness. If the selected hospital cannot complete the research study, it will be replaced by an eligible hospital of the same capacity in the same economic-geographic region stratum. The status of hospitals in each cluster will not be shared with hospitals outside the cohort.

**Participants/Study Duration**	Approximately 7644 participants within 2 years.
**Study Population**	AIS patients within 6 hours of syptom onset.
**Intervention**	We developed the comprehensive intervention ***"STEP"*** (Strategies, Toolkit, Exploration, Paradigm) to promote the reconstruction of workflow in stroke centres and shorten in-hospital delay of reperfusion treatment for AIS patients. The main intervention objects contain managers, physicians (including emergency doctors, radiologists, neurologists or vascular neurologists, interventionists or neurosurgeons, anaesthesiologists) and nurses in the participating hospitals.
**Evaluation Period for Outcome Events**	Clinical Events outcomes will be measured at all participating hospitals prior to the intervention and whenever a new hospital ('step') receives the intervention. At each step there will be a comparison of clinical outcomes between hospitals and those not receiving it. Evaluation period will continue till all hospitals receive the intervention.
**Outcomes assessment**	Primary outcome:<br>▪ the rate of adherence to reperfusion treatment, including IVT for eligible patients who arrived within 3.5hrs or EVT for eligible patients who arrived within 4.5hrs.<br>Secondary outcomes:<br>▪ the proportion of patients receiving IVT in those who arrived in the hospital within 3.5hrs after onset;<br>▪ the proportion of patients receiving EVT in those who arrived at the hospital within 4.5hrs after onset;<br>▪ door-to-needle time (DNT) within ;<br>▪ door-to-puncture time (DPT);<br>▪ in-hospital mortality;<br>▪ 3-month disability as measured by mRS above 2.
**Statistical Methodology**	All efficacy analyses will use the intention-to-treat principle. Continuous variables will be presented as the means and standard deviations or medians with interquartile ranges, and categorical variables are presented as counts and percentages. The baseline characteristics will be compared by analysis of variance or Kruskal-Wallis test for continuous variables and $\chi 2$ test or Fisher exact test for categorical variables. The primary outcome will be analysed using a mixed-effects logistic

	regression with a random effect for the cluster (hospital) and a fixed time effect for every step. For categorical secondary outcomes, data will be analysed using the same strategy for the primary outcome. For continuous secondary outcomes, mixed-effects linear regression with a random effect for the cluster (hospital) and a fixed time effect for the step will be used. All tests will be performed in SAS software version 9.4 (SAS Institute, Cary, NC).
**Date of Protocol**	May 18, 2018

Table of Contents

1. **BACKGROUD** 6
2. **Study Design** 7
3. **RANDOMIZATION OF HOSPITALS** 8
4. **DURATION OF INTERVENTION** 8
5. **PARTICIPATING HOSPITALS AND PATIENTS** 8
6. **INTERVENTION** 10
7. **ORGANIZATIONAL STRUCTURE** 11
8. **OUTCOMES** 12
   - 8.1 Primary Outcomes 12
   - 8.2 Secondary Outcomes 12
9. **SAMPLE ESTIMATION** 12
10. **DATA ACCESS** 12
11. **ANALYTIC PLAN** 13

REFERENCES 14

1. BACKGROUD

Stroke is the leading cause of death and disability worldwide.[1] As the most populous country in the world with a rapidly aging population, China faces an immense healthcare challenge due to stroke. [2] The burden of stroke in China has risen steeply in the past three decades, with disability-adjusted life-years increasing by 24.4% between 1990 and 2017.[3] One such way to mitigate this burden is through reperfusion therapy.

Reperfusion therapy is the most effective treatment for acute ischemic stroke (AIS).[2] Several large randomized controlled trials have shown that intravenous recombinant tissue plasminogen activator (IV rtPA) and endovascular thrombectomy (EVT) significantly improve 3-month functional outcomes.[3-9] Intravenous thrombolysis (IVT) for eligible patients within 4.5 hours after onset and EVT for eligible patients within 6 hours after onset were recommended by the American Heart Association /American Stroke Association (AHA/ASA) and Chinese Stroke Association (CSA), respectively.[10, 11] However, adherence to IVT therapy in China was only 18.3% in 2012, which was still far from the adherence reported by the Get With The Guidelines-Stroke (GWTG-Stroke) Study in the United States (72.8%)[12, 13]. Additionally, the proportion of eligible patients undergoing EVT within 6 hours was only 2.17% in data from the China Stroke Center Alliance (CSCA) (unpublished) in March 2018.[14] Given the low adherence to both IVT and EVT, there is an urgent need to develop tailored strategies to increase adherence for eligible patients within the guideline-recommended time window.

As an effective quality improvement program for reperfusion therapy, “Target: Stroke” has achieved tremendous success in improving in-hospital healthcare quality of AIS, especially in shortening door-to-needle time and increasing the thrombolysis rate.[15] Accordingly, multidimensional quality improvement initiatives targeting reperfusion therapy, including clinical decision support tools, facilitating hospital participation, and sharing best practices, which may be effective in improving

adherence to evidence-based performance measures. However, there is a lack of evidence for effective interventions in low- and middle-income countries.

We developed the comprehensive intervention ***"STEP"*** (Strategies, Toolkit, Exploration, Paradigm) to promote the reconstruction of workflow in stroke centres and to shorten in-hospital delay of reperfusion treatment for AIS patients. We hypothesize that the STEP intervention will increase adherence to IVT or EVT and improve 3-month clinical outcomes in patients with AIS in China. In addition, we seek to evaluate the influence of different intervention durations on adherence to reperfusion therapy guidelines.

2. Study Design

The Improve Acute Reperfusion Treatment Quality for Stroke in China (IMPROVE: Stroke Care in China) trial is a stepped wedge cluster randomized trial to assess the effectiveness of the STEP intervention on adherence to guideline-recommended reperfusion therapy for AIS patients in China. Using the stepped wedge cluster randomized trial design,[16] participating hospitals will be randomized to three cohorts for different predefined steps to receiving the intervention. After a 6-month initial period, the STEP intervention will be implemented in cohort 1 through the end of the trial. The intervention will be implemented in cohort 2 at 13 months through the end of the trial, and in cohort 3 at 19 months through the end of the trial. There will not be a transition period for any of the cohorts (Figure 1). All patients in each cohort will be included consecutively and undergo a 3-month follow-up period. The protocol of this study was approved by the central institutional review board at Beijing Tiantan Hospital, Capital Medical University and participating hospitals, and written informed consent will be obtained prior to enrollment. This trial has been registered online (www.clinicaltrials.gov identifier NCT003578107).

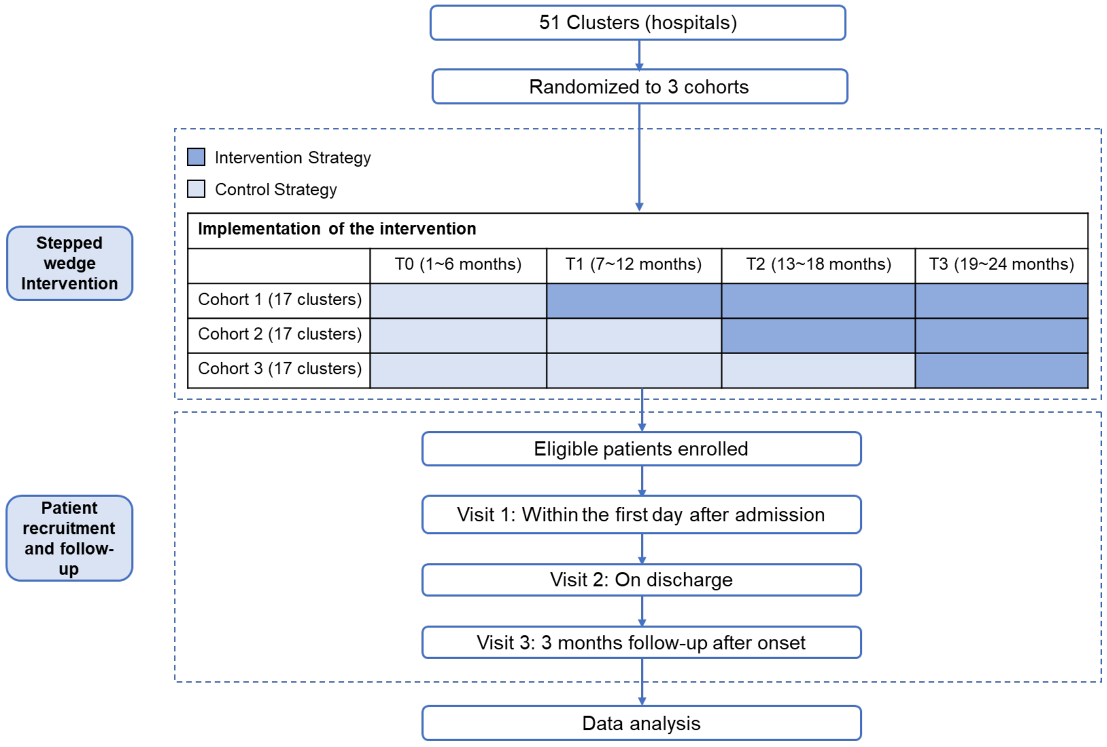

Figure 1. The flow chart of the IMPROVE trial

3. RANDOMIZATION OF HOSPITALS

Cluster randomization at the hospital level will be performed centrally using a computer-generated random number sequence that will only be known by two independent biostatisticians (YS Pan and HQ Gu). Hospital location and grade will be matched during the randomization to minimize imbalances throughout the trial. A confirmation letter will be sent to all the hospitals in a particular cohort one month before the implementation of the intervention to ensure preparedness. If the selected hospital cannot complete the research study, it will be replaced by an eligible hospital of the same capacity in the same economic-geographic region stratum. The status of hospitals in each cluster will not be shared with hospitals outside the cohort.

4. DURATION OF INTERVENTION

In cohort 1, the STEP intervention will be implemented for 18 months; in cohort 2, for 12 months and in cohort 3, for 6 months.

5. PARTICIPATING HOSPITALS AND PATIENTS

Participating hospitals will be enrolled from mainland China according to the

economic-geographic regions: Eastern, Central, and Western. To ensure the diversity and representativeness of the included clusters, the number of hospitals enrolled from each stratum will be proportional to the number of secondary and tertiary hospitals in each region. Each participating hospital will designate a principal investigator and a physician or nurse as the quality improvement research coordinator. The inclusion and exclusion criteria of participating hospitals are shown in Table. A total of 51 secondary or tertiary hospitals (clusters) were randomly selected from 75 voluntary hospitals which located in 29 provinces, autonomous regions or municipalities (Anhui, Beijing, Fujian, Gansu, Guangdong, Guangxi, Guizhou, Hainan, Hebei, Henan, Heilongjiang, Hubei, Hunan, Inner Mongolia, Jilin, Jiangsu, Jiangxi, Liaoning, Ningxia, Qinghai, Shandong, Shanxi, Shannxi, Sichuan, Tianjin, Xinjiang, Yunnan, Zhejiang, and Chongqing).

Table. The specific hospital inclusion and exclusion criteria.

Inclusion criteria
1) Voluntary;
2) Secondary or tertiary public hospitals with an emergency department and neurologic wards that admit patients with AIS;
3) Has a 24*7 on-call stroke team;
4) Has the capacity for IVT and/or EVT;
5) Implemented at least 10 patients' IVT and/or EVT during the last year;
6) Admitted at least 5 patients within 6 hours after onset each month;
7) Desire to improve treatment workflow of AIS;
8) Good cooperation among neurology department, emergency department, interventional department, neurosurgery department, laboratory and radiology department.
**Exclusion criteria**
1) Refusal to participate;
2) Primary or private hospitals;
3) Hospitals participated in other stroke care quality improvement projects or related

clinical trials.

Patients eligible for inclusion are those over 18 years of age, present with symptoms of AIS and confirmed by CT and/or MRI, and arrive at the hospital within 6 hours after stroke onset. Patients diagnosed with transient ischemic attack (TIA), hemorrhagic stroke and non-cerebrovascular diseases will not be included in this trial. Informed consent will be obtained from patients or their proxies prior to enrollment.

6. INTERVENTION

Strategies for the ***STEP*** intervention will be performed to promote the reconstruction of workflow in stroke centres and to shorten in-hospital delay of reperfusion treatment for AIS patients. The main intervention objects contain managers, physicians (including emergency doctors, radiologists, neurologists or vascular neurologists, interventionists or neurosurgeons, anaesthesiologists) and nurses in the participating hospitals.

- One group of best practice ***S*****trategies** is based on "Target: Stroke" and aims to optimize the Green Channel.[15] It includes hospital pre-notification by the emergency medical system (120/999 in China), rapid triage, a single call activation of the stroke team, a designated staff supporting the whole process (including taking patients to CT scan, helping to pay for therapy, etc.), a patient-specific chart with time record, a principle of CT scan priority for stroke patients, a stroke team that directly goes to CT room, rapid acquisition and interpretation of brain imaging, stroke first for laboratory testing (including point of care testing if indicated), rtPA regularly stored in the thrombolysis room of emergency department (ER), receiving IVT as soon as possible, and some other written documents from CSA or National Center for Healthcare Quality Management in Neurological Diseases to receive support from hospital management.
- One integrated ***T*****oolkit** for AIS treatment is presented as a handbook and designed according to the AHA/ASA and CSA guidelines for AIS management.[10, 11] It is integrated with the key tools that have been used and

updated in the Green Channel of Beijing Tiantan Hospital, Capital Medical University. It includes the rapid triage protocol, clinical pathways, recommended time intervals, brain CT/MRI interpretation protocol, indications and contraindications of IVT/EVT, stroke-specific order sets, rt-PA application process, calculated dosage by different weights, peri-IVT/EVT management, stroke scales (National Institute of Health Stroke Scale [NIHSS] and modified Rankin Scale [mRS]), updated guidelines, informed consent for IVT/EVT and relevant contact information.

- The ***E*****xploration** component provides guidance by experts on improving the intervention. Given the experience of improving clinical practice in Beijing Tiantan Hospital, Capital Medical University, a regular point-to-point guidance to hospitals will be provided by experienced experts through teleconferences when each cohort crosses from the control to the interventional steps. Some remote technical trainings and workshops will be held as well.

The ***P*****aradigm** of the feedback system serves to make a Play-Do-Study-Act (PDSA) circle. Weekly data feedback will be provided to sites through the online data feedback platform, WeChat App, telephone, or email by the assigned quality coordinator of this trial. A weekly or biweekly meeting will be encouraged in hospitals receiving the intervention to analyze the reasons for in-hospital delays in receiving IVT/EVT or not receiving IVT/EVT at all. A biweekly or monthly communication with the site contact person will be performed and some improvement strategies will be recommended by the quality coordinator., On-site monitoring will be conducted on the establishment and improvement of IVT/EVT procedures in each hospital.

7. ORGANIZATIONAL STRUCTURE

This study is administered by an international academic steering committee composed of co-principle investigators and members. This committee is responsible for the study design, research protocol, academic support and phased progress by Senior Management Group (SMG) meetings. The quality improvement committee is

composed of clinical and management experts and responsible for supervising the current adherence of IVT/EVT, determining measures to improve the adherent rate and providing technical support for workshops, remote training, and point-to-point guidance to the hospitals receiving the intervention. The executive committee is composed of senior experts from the international academic steering committee. The purpose of this committee is to assess the progress and safety of the study and make decisions regarding early termination, modification, or continuation of the trial.

8. OUTCOMES

8.1 Primary Outcomes

The primary outcome is the rate of adherence to reperfusion treatment, including IVT for eligible patients who arrived within 3.5hrs or EVT for eligible patients who arrived within 4.5hrs.

8.2 Secondary Outcomes

The secondary outcomes include the proportion of patients receiving IVT in those who arrived in the hospital within 3.5hrs after onset; the proportion of patients receiving EVT in those who arrived at the hospital within 4.5hrs after onset; door-to-needle time (DNT) within 60 minutes; door-to-puncture time (DPT) within 90 minutes; in-hospital mortality; and 3-month disability as measured by mRS above 2.

9. SAMPLE ESTIMATION

Based on the CSCA data up to March 2018, we assume that the baseline adherent rate of IVT or EVT for eligible patients within 6 hours is approximately 19%. As such, 51 participating hospitals (17 for each cluster) enrolling 31 patients per time period (6 months) per site will be required to detect a relatively 30% improvement (from 19% to 25%) with 90% power, alpha =0.05, and an intracluster correlation coefficient (ICC) of 0.03. That would be 6324 patients totally. We increased the sample size to 7644, allowing a follow-up loss rate up to 17% at 3-month mRS evaluation.

10.DATA ACCESS

The variables in the case report form (CRF) of this trial are mainly sourced from the China National Stroke Registries and the GWTG-Stroke, and are confirmed by the executive committee.[13, 17] All variables of baseline data on demographics, medical history, vascular risk factors, pre-stroke mRS, key time points (time of onset, to door, to image, to needle, to puncture, and to recanalization), NIHSS score on admission, imaging data and information of IVT/EVT are obtained at admission by trained research coordinators in each site. The second follow-up will be performed at discharge to collect information about the final diagnosis, NIHSS score and mRS at discharge, aetiological classification, in-hospital complications, discharge secondary prevention medications, hospitalization expenses and other in-hospital medical treatments. The aetiological classification will use the Trial of Org 10172 in Acute Stroke Treatment (TOAST) criteria.[18] A centralized follow-up interview by telephone will be conducted at 3 month after onset to collect data on current mRS, medication use and compliance, stroke recurrence and all-cause mortality. All the interviewers will be blinded to which cluster patients are in.

All information of included patients will be collected and uploaded by a web-based Patient Management Tool (GaiDe, Inc., Beijing, China) at each site. To be compliant with the national privacy standards, all data will be encrypted and transmitted to the China National Clinical Research Center for Neurological Diseases, which will serve as the data analysis and feedback centre. If crossing from control to the interventional step, all feedback data of current adherent rate can be checked by each participating hospital online; all data will be sent to the principal investigator of each hospital via WeChat App and email.

11. ANALYTIC PLAN

All efficacy analyses will use the intention-to-treat principle.[19] Continuous variables are presented as the means and standard deviations or medians with interquartile ranges, and categorical variables are presented as counts and percentages. The baseline characteristics will be compared by analysis of variance or Kruskal-Wallis test for continuous variables and $\chi 2$ test or Fisher exact test for categorical variables.

The primary outcome will be analysed using a mixed-effects logistic regression with a random effect for the cluster (hospital) and a fixed time effect for every step. For categorical secondary outcomes, data will be analysed using the same strategy for the primary outcome. For continuous secondary outcomes, mixed-effects linear regression with a random effect for the cluster (hospital) and a fixed time effect for the step will be used. All tests will be performed in SAS software version 9.4 (SAS Institute, Cary, NC).

REFERENCES

1. Global burden of 369 diseases and injuries in 204 countries and territories, 1990-2019: A systematic analysis for the global burden of disease study 2019. *Lancet (London, England)*. 2020;396:1204-1222
2. Campbell BCV, Khatri P. Stroke. *Lancet (London, England)*. 2020;396:129-142
3. Tissue plasminogen activator for acute ischemic stroke. *N Engl J Med*. 1995;333:1581-1587
4. Hacke W, Kaste M, Bluhmki E, Brozman M, Davalos A, Guidetti D, et al. Thrombolysis with alteplase 3 to 4.5 hours after acute ischemic stroke. *N Engl J Med*. 2008;359:1317-1329
5. Berkhemer OA, Fransen PS, Beumer D, van den Berg LA, Lingsma HF, Yoo AJ, et al. A randomized trial of intraarterial treatment for acute ischemic stroke. *N Engl J Med*. 2015;372:11-20
6. Campbell BC, Mitchell PJ, Kleinig TJ, Dewey HM, Churilov L, Yassi N, et al. Endovascular therapy for ischemic stroke with perfusion-imaging selection. *N Engl J Med*. 2015;372:1009-1018
7. Goyal M, Demchuk AM, Menon BK, Eesa M, Rempel JL, Thornton J, et al. Randomized assessment of rapid endovascular treatment of ischemic stroke. *N Engl J Med*. 2015;372:1019-1030
8. Jovin TG, Chamorro A, Cobo E, de Miquel MA, Molina CA, Rovira A, et al. Thrombectomy within 8 hours after symptom onset in ischemic stroke. *N Engl J Med*. 2015;372:2296-2306
9. Saver JL, Goyal M, Bonafe A, Diener HC, Levy EI, Pereira VM, et al. Stent-retriever

thrombectomy after intravenous t-pa vs. T-pa alone in stroke. *N Engl J Med*. 2015;372:2285-2295

10. Powers WJ, Rabinstein AA, Ackerson T, Adeoye OM, Bambakidis NC, Becker K, et al. 2018 guidelines for the early management of patients with acute ischemic stroke: A guideline for healthcare professionals from the american heart association/american stroke association. *Stroke*. 2018;49:e46-e110
11. Association CS. 2018 guideline for endovascular treatment in acute ischemic stroke patients. *Chin J Stroke*. 2018;13:706-729
12. Li Z, Wang C, Zhao X, Liu L, Wang C, Li H, et al. Substantial progress yet significant opportunity for improvement in stroke care in china. *Stroke*. 2016;47:2843-2849
13. Schwamm LH, Fonarow GC, Reeves MJ, Pan W, Frankel MR, Smith EE, et al. Get with the guidelines-stroke is associated with sustained improvement in care for patients hospitalized with acute stroke or transient ischemic attack. *Circulation*. 2009;119:107-115
14. Wang Y, Li Z, Wang Y, Zhao X, Liu L, Yang X, et al. Chinese stroke center alliance: A national effort to improve healthcare quality for acute stroke and transient ischaemic attack: Rationale, design and preliminary findings. *Stroke Vasc Neurol*. 2018;3:256-262
15. Fonarow GC, Zhao X, Smith EE, Saver JL, Reeves MJ, Bhatt DL, et al. Door-to-needle times for tissue plasminogen activator administration and clinical outcomes in acute ischemic stroke before and after a quality improvement initiative. *JAMA*. 2014;311:1632-1640
16. Hemming K, Haines TP, Chilton PJ, Girling AJ, Lilford RJ. The stepped wedge cluster randomised trial: Rationale, design, analysis, and reporting. *BMJ*. 2015;350:h391
17. Wang Y, Jing J, Meng X, Pan Y, Wang Y, Zhao X, et al. The third china national stroke registry (cnsr-iii) for patients with acute ischaemic stroke or transient ischaemic attack: Design, rationale and baseline patient characteristics. *Stroke Vasc Neurol*. 2019;4:158-164
18. Adams HP, Jr., Bendixen BH, Kappelle LJ, Biller J, Love BB, Gordon DL, et al. Classification of subtype of acute ischemic stroke. Definitions for use in a multicenter clinical trial. Toast. Trial of org 10172 in acute stroke treatment. *Stroke*. 1993;24:35-41
19. Jo B, Asparouhov T, Muthén BO. Intention-to-treat analysis in cluster randomized trials with noncompliance. *Stat Med*. 2008;27:5565-5577

Improve Acute Reperfusion Treatment Quality for Stroke: IMPROVE Stroke Care in China

Statistical Analysis Plan

Version: 1.1

Date: July 1, 2021

by: Hong-Qiu Gu & Chun-Juan Wang

In signing this document, I am confirming that I have reviewed and approved the SAP for IMPROVE Stroke Care in China Trail.

Approved by	Signature	Date
Prof. Yong Jiang		
Prof. Hao Li		
Prof. Zi-Xiao Li		
Prof. Yong-Jun Wang		

1 Introduction

The purpose of the statistical analysis plan is to describe key components of the Improve Acute Reperfusion Treatment Quality for Stroke in China (IMPROVE Stroke Care in China).

The IMPROVE Stroke Care trial is a cluster-randomized, stepped-wedge, pragmatic clinical trial in which 51 hospitals (clusters) in China were randomized to receive a targeted quality improvement intervention at 1 of 3 predefined, 6-month periods over an 18-month period between January 1, 2019, and June 30, 2020, after a 6-month period of usual care.

2 Study objectives

This study aims to investigate the efficacy of a targeted quality improvement intervention on adherence to guideline-recommended reperfusion therapy for patients with acute ischemic stroke in China

2.1 Primary objective

To determine if the targeted quality improvement intervention increases the administration of guideline-recommended reperfusion therapy for patients with acute ischemic stroke within 6 hours of symptom onset.

2.2 Secondary objective

To determine if the targeted quality improvement intervention:

- Increases the rate of IV rt-PA within 4.5hrs of symptom onset among eligible patients

- Increases the rate of EVT within 6hrs of symptom onset among eligible participants
- Increases the proportion of patients with door-to-needle time (DNT) within 60 min among participants who received IV-rtPA
- Increases the proportion of patients with door-to-puncture time (DPT) within 90 min among participants who received EVT
- Reduces in-hospital mortality
- Reduces 3-month disability as measured by the mRS above 2

3 Study design

The IMPROVE Stroke Care trial is a cluster-randomized, stepped-wedge, pragmatic clinical trial in which 51 hospitals (clusters) in China were randomized to receive a targeted quality improvement intervention at 1 of 3 predefined, 6-month periods over an 18-month period between January 1, 2019, and June 30, 2020, after a 6-month period of usual care.

3.1 Hospitals and Study Participants

A total of 51 secondary or tertiary hospitals (clusters) located in 29 provinces, autonomous regions or municipalities were selected voluntarily from the CSCA hospitals.

To ensure the diversity and representativeness of the included hospitals, hospitals were enrolled with the consideration of economic-geographic regions (Eastern, Central, and Western) and hospital-level (secondary or tertiary). To be eligible, hospitals should be secondary or tertiary public hospitals with an emergency department and neurologic wards, has a 24*7 on-call stroke team, has the capacity for IVT or EVT, at least administered IVT or EVT to 10 patients during the whole year before enrollment, and at least 5 patients were admitted within 6 hours after stroke onset per month. All hospitals must have a desire to improve the workflow of

treating acute ischemic stroke and have good collaboration among the multidisciplinary team. Hospitals that participated in other quality improvement projects were all excluded.

Patients were consecutively enrolled if they were over 18 years of age, presented with symptoms of acute ischemic stroke and confirmed by computed tomography (CT) or magnetic resonance imaging (MRI), and arrived at the hospital within 6 hours of symptoms onset. Patients diagnosed with other cerebrovascular diseases, such as transient ischemic attack, hemorrhagic stroke, cerebral venous sinus thrombosis, or noncerebrovascular diseases, were excluded.

3.2 Randomization

The study biostatistician at the central coordinating center (Beijing Tiantan Hospital) will centrally randomize the hospital(cluster) using a computer-generated randomization sequence and maintain the privacy of randomization records prior to the beginning of the data collection on a password-protected secure server. A total of 17 hospitals would be randomly assigned to one of three sequences (cohorts) with the consideration of hospital location and level to minimize potential imbalance between the intervention and control periods as far as possible. The other members of the study team and the selected sites were informed that they would cross over to the intervention period one month before each of the predefined steps to maintain allocation concealment while aiding in training logistics.

3.3 Intervention and Training

A targeted quality improvement intervention, named “STEP” (Strategies, Toolkit, Exploration, and Paradigm), was developed to promote the reconstruction of

workflow in stroke centers and to shorten in-hospital delay of reperfusion treatment for acute ischemic stroke.

To be brief, “STEP” means (1) Strategies: a group of best practice strategies based on “Target: Stroke” aimed to optimize the Green Channel, including hospital prenotification, rapid triage, a single call activation of the stroke team, a designated staff supporting the whole process of reperfusion therapy, a patient-specific chart with time record, a principle of CT scan priority for patients with acute ischemic stroke, a stroke team that directly goes to CT room, rapid acquisition and interpretation of brain imaging, stroke first for laboratory testing, rtPA regularly stored in the thrombolysis room of emergency department, receiving IVT/EVT as soon as possible, and some other written documents from China Stroke Association (CSA) or National Center for Healthcare Quality Management in Neurological Diseases to receive administrative support from hospital management; (2) Toolkit: an integrated toolkit for acute ischemic stroke treatment presented as a handbook and designed according to the AHA/ASA and CSA guidelines, including triage protocol, clinical pathways, recommended time intervals, brain CT/MRI interpretation protocol, indications and contraindications of IVT/EVT, stroke-specific order sets, rt-PA application process, calculated dosage by different weights, peri-IVT/EVT management, stroke scales, updated guidelines, informed consent for IVT/EVT and relevant contact information; (3) Exploration: a new interventional strategy was explored by providing expertise guidance to hopitals entering into the intervention stage. In this exploration, a group of experienced experts from Beijing Titantan Hospital, Capital Medical University was invited to join a regular teleconference to give professional or technical guidance or training that was specific to the hospital in need when each cohort crosses from the control to the interventional steps. This was a much-localized strategy in accordance with the medical system in China; (4) Paradigm: the paradigm of the feedback system served to make a Plan-Do-Study-Act (PDSA) circle, including on-site monitoring.

3.4 Outcomes measures

3.4.1 Primary Outcomes

The primary outcome was the rate of reperfusion treatments. It was defined as a composite outcome of IVT for eligible participants who arrived within 3.5hrs of symptom onset and EVT for eligible participants who arrived within 4.5hrs of symptom onset.

3.4.2 Secondary Outcomes

The secondary outcomes included:

- the rate of IV rt-PA among eligible patients who arrived within 3.5hrs of symptom onset;
- the rate of EVT among eligible patients who arrived within 4.5hrs of symptom onset;
- the proportion of patients with door-to-needle time (DNT) within 60 min among participants who received IV-rtPA;
- the proportion of patients with door-to-puncture time (DPT) within 90 min among participants who received EVT;
- in-hospital mortality;
- and 3-month disability as measured by the mRS above 2.

4 Statistical Analysis

4.1 General Principles

Data analyses will be performed at the central coordinating center (Beijing Tiantan Hospital). Only de-identified data will be analyzed by the team at Beijing Tiantan Hospital. This SAP will be finalized prior to the database lock. The statistical analyses will be performed using SAS Version 9.4.

All analyses will be performed on the principle of "intension- to treat". Baseline characteristics at the individual level will be summarized by mean (SD) or median (interquartile ranges [IQR]) for continuous variables and counts with percentages for categorical variables. Baseline characteristics between intervention and control participants will be compared using crude differences and 95% confidence intervals.

Mixed-effects logistic regression models with a random effect for the cluster (hospital) and a fixed time effect for every 6-month period will be used to assess the effect of the intervention. If the models are not converging, generalized estimating equation (GEE) models with consideration for the clustering within hospitals and a fixed time effect for the period will be fitted.

4.2 Missing data handling

Missing data will be kept to a minimum, as the nature of the trial and primary outcomes are collected during hospitalization. However, if data on the 90d follow-up assessment is missing, we will perform sensitivity analyses using complete case analysis and multiple imputations to check the roubst of our analyses. Results from analyses based on multiple imputations would be pooled according to Rubin's rules.[1]

4.3 Primary outcome analyses

The primary outcome was the rate of reperfusion treatments. The differences in rates of reperfusion treatments will be modelled using mixed effect logistic regression with random cluster (hospital) effects and a fixed time effect for the period (6 months). If the models failed to converge, GEE logistic models would be used instead.

To check the robustness of our primary analysis, we will also model the outcomes with adjustment for baseline imbalanced covariates (covariates-adjusted analysis). In all of the above analyses, we will reporte the absolute effect measure risk difference (RD) and 95% CI estimated from a binomial regression model with the link function set to identity, in addition to the relative effect measure OR and 95% CI.[2]

4.4 Secondary outcome analyses

The secondary outcomes included the rate of IV rt-PA among eligible patients who arrived within 3.5hrs of symptom onset; the rate of EVT among eligible patients who arrived within 4.5hrs of symptom onset; the proportion of patients with door-to-needle time (DNT) within 60 min among participant received IV-rtPA; the proportion of patients with door-to-puncture time (DPT) within 90 min among participant received EVT; in-hospital mortality; and 3-month disability as measured by the mRS above 2.

All secondary outcomes will be analzyed using the same strategy with the primary outcome.

4.5 Sensitivity analyses

A seireis sensitivy analyses will be performed, including

- covariates (covariates-adjusted analysis) for the primary or secondary outcomes.
- subgroup analyses by age (<65, >=65 years), sex, and hospital level (secondary or tertiary) in the above-mentioned primary and covariable-adjusted analysis.

- Complete case analysis and multiple imputation analyses for 90d disability
- And other sensitivity analysis

5 References

1. Rubin DB. *Multiple Imputation for Nonresponse in Surveys.* Hoboken: Wiley; 1987.
2. Pedroza C, Truong VT. Performance of models for estimating absolute risk difference in multicenter trials with binary outcome. *BMC Med Res Methodol.* 2016;16(1):113.
