# Supplementary material for: Effectiveness of a Quality Improvement Intervention on Reperfusion Treatment for Patients With Acute Ischemic Stroke: A Stepped-Wedge Cluster Randomized Clinical Trial
Source: JAMA Netw Open. 2023 Jun 2;6(6):e2316465. doi: 10.1001/jamanetworkopen.2023.16465 (PMC10238948; doi:10.1001/jamanetworkopen.2023.16465)
Supplement: Supplement 2. — eMethods. eTable 1. Baseline Characteristics of the IMPROVE Stroke Care in China Patients and Hospitals Between Included and Excluded Patients eTable 2. Baseline Characteristics of the IMPROVE Stroke Care China Patients and Hospitals Among Patients With and Without Values of mRS at 90 Days eTable 3. Results of Disability at 90 Days From Multiple Imputations eTable 4. Subgroup Analysis for the Effect of Intervention on Reperfusion Treatment for Other Secondary Outcomes eTable 5. Baseline Characteristics of the IMPROVE Stroke Care in China Patients and Hospitals by Reperfusion eTable 6. Results of Disability at 90 Days From Raw Data eFigure 1. The Design of “STEP” Intervention eFigure 2. Reperfusion Therapy in the Chinese Center Association Alliance Hospitals Not Participating in the IMPROVE Stroke Care in China Trial eFigure 3. Distribution of Door-to-Puncture Time in the IMPROVE Stroke Care In China Trial [file jamanetwopen-e2316465-s002.pdf]

# Supplementary Online Content

Wang CJ, Gu HQ, Zong LX, et al; IMPROVE Stroke Care in China investigators. Effectiveness of a quality improvement intervention on reperfusion treatment for patients with acute ischemic stroke: a stepped-wedge cluster randomized clinical trial. *JAMA Netw Open*. 2023;6(6):e2316465. doi:10.1001/jamanetworkopen.2023.16465

**eMethods**
**eTable 1.** Baseline Characteristics of the IMPROVE Stroke Care in China Patients and Hospitals Between Included and Excluded Patients
**eTable 2.** Baseline Characteristics of the IMPROVE Stroke Care in China Patients and Hospitals Among Patients With and Without Values of mRS at 90 Days
**eTable 3.** Results of Disability at 90 Days From Multiple Imputations
**eTable 4.** Subgroup Analysis for the Effect of Intervention on Reperfusion Treatment for Other Secondary Outcomes
**eTable 5.** Baseline Characteristics of the IMPROVE Stroke Care in China Patients and Hospitals by Reperfusion
**eTable 6.** Results of Disability at 90 Days From Raw Data
**eFigure 1.** The Design of "STEP" Intervention
**eFigue 2.** Reperfusion Therapy in the Chinese Center Association Alliance Hospitals Not Participating in the IMPROVE Stroke Care in China Trial
**eFigure 3.** Distribution of Door-to-Puncture Time in the IMPROVE Stroke Care In China Trial

This supplementary material has been provided by the authors to give readers additional information about their work.

© 2023 Wang CJ et al. *JAMA Network Open.*

**eMethods**

The "STEP" means (1) **S**trategies, namely intervention characteristics: a group of best practice strategies developed from the "Target: Stroke Initiative" according to the outer and inner settings of hospitals in China. It aimed to construct the Green Channel for reperfusion therapy and to optimize the workflow of rescuing patients in stroke centers, including hospital prenotification, rapid triage, a single call activation of the stroke team, a designated staff supporting the whole process of reperfusion therapy, a patient-specific chart with time records, a principle of prioritizing CT scans for patients with acute ischemic stroke, a stroke team that goes directly to the CT room, the rapid acquisition and interpretation of brain imaging, prioritizing laboratory testing for stroke patients, rtPA regularly stored in the thrombolysis room of the emergency department and providing IVT/EVT as soon as possible. All related staff, including the administrators, physicians, nurses, and someone supportive from different departments, were all trained at the beginning of the first month when entering into the intervention phase. Some other written documents from the China Stroke Association (CSA) or National Center for Healthcare Quality Management in Neurological Diseases were provided to receive administrative support from hospital management because the multidisciplinary organization and coordination mainly relied on the head of the administration (e.g., the president or vice-president) in public hospitals in the health settings of China; (2) **T**oolkit: an integrated toolkit for acute ischemic stroke treatment was designed according to the AHA/ASA and CSA guidelines. It is presented as a handbook and includes triage protocol, clinical pathways, recommended time intervals for different departments, brain CT/MRI interpretation

© 2023 Wang CJ et al. *JAMA Network Open.*

protocols, indications and contraindications of IVT/EVT, stroke-specific order sets, rt-PA application process, calculated dosage by different weights, peri-IVT/EVT management, frequently used stroke scales, updated guidelines for acute stroke management and relevant contact information (i.e., telephone number) of all staff working in the whole process of reperfusion therapy. Considering the characteristics of each individual, informed consent forms with full consideration of medical humanities for IVT/EVT were implemented to get trust from patients or their proxies; (3) **E**xploration: a new intervention strategy was explored by providing expert guidance to hospitals entering into the intervention stage to reduce the unequal distribution of high-quality health resources. In this exploration, a group of experienced experts from Beijing Tiantan Hospital, Capital Medical University, was invited to join a regular teleconference to provide professional or technical guidance or training that was specific to the hospital in need 1:1 when each cohort crossed from the control step to the interventional step. This was a much more localized strategy in accordance with the medical system in China. (4) **P**aradigm: the paradigm of the feedback system served to make a Plan-Do-Study-Act (PDSA) circle, including on-site monitoring. All information of included patients, especially those related to reperfusion therapy, was collected and uploaded through a web-based case report form which additionally contained a Patient Management Tool (GaiDe, Inc, Beijing, China) for feedback. After crossing from the control to the intervention stage, all feedback data of the current adherent rate of reperfusion therapy would be sent to each site weekly, and the participating hospital could check it online through an authorized username and password. Meanwhile, all these data reports would be sent to the principal investigator and the

© 2023 Wang CJ et al. *JAMA Network Open.*

administrator of reperfusion therapy in each hospital via WeChat App and email. An improvement meeting was required weekly or biweekly within sites. Onsite audits or teleconference communication were conducted by month to find the existing problems during the intervention, and the steering committee would make a way to solve them. The online and offline data checks of all patients were performed within two weeks after inclusion. The interventions were administered to administrators, physicians, and nurses in the participating hospitals but not to the individual patients.

© 2023 Wang CJ et al. *JAMA Network Open.*

**eTable 1.** Baseline Characteristics Of the IMPROVE Stroke Care In China Patients And Hospitals Between Included and Excluded Patients.

| Characteristics | Included (N=12132 [76.4%]) | Excluded (N=3756 [23.6%]) | Difference (95% CI) |
|---|---|---|---|
| **Patient characteristics** | | | |
| Demographic | | | |
| Age, mean (SD), y | 66.1±12.1 | 66.1±11.9 | -0.1 (-0.5 to 0.4) |
| Male | 7759 (64.0) | 2433 (64.8) | -0.8 (-2.6 to 0.9) |
| BMI, mean (SD), kg/m2 | 23.8±3.8 | 24.0±4.7 | -0.2 (-0.4 to -0.0) |
| NIHSS at admission | 5.0 (2.0 – 10.0) | 4.0 (2.0 – 7.0) | -1.0 (-1.0 to -1.0) |
| Current smoking | 3238 (26.7) | 1038 (27.6) | -0.9 (-2.6 to 0.7) |
| Drinking | 2993 (24.7) | 939 (25.0) | -0.3 (-1.9 to 1.3) |
| Medical history | | | |
| Stroke or TIA | 3445 (28.4) | 1193 (31.8) | -3.4 (-5.1 to -1.7) |
| Hypertension | 7457 (61.5) | 2328 (62.0) | -0.5 (-2.3 to 1.3) |
| Diabetes | 2401 (19.8) | 831 (22.1) | -2.3 (-3.8 to -0.8) |
| Dyslipidemia | 725 (6.0) | 199 (5.3) | 0.7 (-0.2 to 1.5) |
| Myocardial infarction | 242 (2.0) | 70 (1.9) | 0.1 (-0.4 to 0.6) |
| Atrial fibrillation | 1107 (9.1) | 275 (7.3) | 1.8 (0.8 to 2.8) |
| Peripheral vascular disease | 165 (1.4) | 28 (0.7) | 0.6 (0.3 to 1.0) |
| Medication history | | | |
| Antiplatelet agent | 2250 (18.5) | 635 (16.9) | 1.6 (0.3 to 3.0) |
| Anticoagulant drugs | 552 (4.5) | 130 (3.5) | 1.1 (0.4 to 1.8) |
| Antihypertensive drugs | 5377 (44.3) | 1667 (44.4) | -0.1 (-1.9 to 1.8) |
| Antidiabetics | 1904 (15.7) | 676 (18.0) | -2.3 (-3.7 to -0.9) |
| Statins | 1961 (16.2) | 528 (14.1) | 2.1 (0.8 to 3.4) |
| Onset to door time in minutes,min | 95.0 (55.0 – 145.0) | 255.0 (227.0 – 298.0) | 161.0 (159.0 to 164.0) |
| **Hospital characteristics** | | | |
| Hospital level | | | |
| Tertiary | 8060 (66.4) | 2591 (69.0) | -2.5 (-4.2 to -0.8) |
| Secondary | 4072 (33.6) | 1165 (31.0) | 2.5 (0.8 to 4.2) |
| Hospital location | | | |
| Eastern | 5574 (45.9) | 1420 (37.8) | 8.1 (6.4 to 9.9) |
| Central | 3664 (30.2) | 1532 (40.8) | -10.6 (-12.3 to -8.8) |
| Western | 2894 (23.9) | 804 (21.4) | 2.5 (0.9 to 3.9) |

Abbreviation: SD, standard deviation; BMI: body mass index; NIHSS, the National Institutes of Health Stroke Scale. TIA, transient ischemic attack.

© 2023 Wang CJ et al. *JAMA Network Open.*

**eTable 2.** Baseline Characteristics of the IMPROVE Stroke Care in China Patients and Hospitals among Patients With and Without Values of mRS at 90 Days.

| Characteristics | With (N=10398 [85.7%]) | Without (N=1734 [14.3%]) | Difference (95% CI) |
|---|---|---|---|
| **Patient characteristics** | | | |
| Demographic | | | |
| Age, mean (SD), y | 65.7±12.0 | 68.4±12.3 | -2.7 (-3.3 to -2.1) |
| Male | 6654 (64.0) | 1105 (63.7) | 0.3 (-2.2 to 2.7) |
| BMI, mean (SD), kg/m2 | 23.8±3.8 | 23.6±3.9 | 0.2 (0.0 to 0.4) |
| NIHSS at admission | 4.0 (2.0 – 9.0) | 6.0 (3.0 – 14.0) | 2.0 (1.0 to 2.0) |
| Current smoking | 2825 (27.2) | 413 (23.8) | 3.4 (1.2 to 5.5) |
| Drinking | 2590 (24.9) | 403 (23.2) | 1.7 (-0.5 to 3.8) |
| Medical history | | | |
| Stroke or TIA | 3013 (29.0) | 432 (24.9) | 4.1 (1.8 to 6.3) |
| Hypertension | 6446 (62.0) | 1011 (58.3) | 3.7 (1.2 to 6.2) |
| Diabetes | 2081 (20.0) | 320 (18.5) | 1.6 (-0.4 to 3.5) |
| Dyslipidemia | 625 (6.0) | 100 (5.8) | 0.2 (-0.9 to 1.4) |
| Myocardial infarction | 195 (1.9) | 47 (2.7) | -0.8 (-1.6 to -0.0) |
| Atrial fibrillation | 871 (8.4) | 236 (13.6) | -5.2 (-6.9 to -3.5) |
| Peripheral vascular disease | 143 (1.4) | 22 (1.3) | 0.1 (-0.5 to 0.7) |
| Medication history | | | |
| Antiplatelet agent | 1947 (18.7) | 303 (17.5) | 1.3 (-0.7 to 3.2) |
| Anticoagulant drugs | 482 (4.6) | 70 (4.0) | 0.6 (-0.4 to 1.6) |
| Antihypertensive drugs | 4653 (44.7) | 724 (41.8) | 3.0 (0.5 to 5.5) |
| Antidiabetics | 1668 (16.0) | 236 (13.6) | 2.4 (0.7 to 4.2) |
| Statins | 1698 (16.3) | 263 (15.2) | 1.2 (-0.7 to 3.0) |
| Onset to door time in minutes,min | 95.0 (55.0 – 145.0) | 91.5 (54.0 – 142.0) | -1.0 (-4.0 to 2.0) |
| **Hospital characteristics** | | | |
| Hospital level | | | |
| Tertiary | 6914 (66.5) | 1146 (66.1) | 0.4 (-2.0 to 2.8) |
| Secondary | 3484 (33.5) | 588 (33.9) | -0.4 (-2.8 to 2.0) |
| Hospital location | | | |
| Eastern | 4798 (46.1) | 776 (44.8) | 1.4 (-1.1 to 3.9) |
| Central | 3272 (31.5) | 392 (22.6) | 8.9 (6.7 to 11.0) |
| Western | 2328 (22.4) | 566 (32.6) | -10.2 (-12.6 to -7.9) |

Abbreviation: SD, standard deviation; BMI: body mass index; NIHSS, the National Institutes of Health Stroke Scale. TIA, transient ischemic attack.

© 2023 Wang CJ et al. *JAMA Network Open.*

**eTable 3.** Results Of Disability At 90 Days From Multiple Imputations.

| | Primary Analysis[a] | | Covariates-Adjusted Analysis[b] | |
|---|---|---|---|---|
| Outcomes | RD (95% CI) | OR (95% CI) | RD (95% CI) | OR (95% CI) |
| Disability at 90d | -1.4 (-5.3 to 2.5) | 0.93 (0.73-1.13) | -0.8 (-4.6 to 3.1) | 0.97 (0.76–1.17) |

Abbreviations: RD, risk difference; and OR, odds ratio.

[a] Results were yielded from generalized estimating equation logistic regression models with adjustment for the hospital clustering effect and temporal period.

[b] Results were yielded from generalized estimating equation logistic regression models with adjustment for the hospital clustering effect, temporal period, and imbalanced baseline covariates (medical history of stroke or transient ischemic attack, medication history of antiplatelet agent and statin, and hospital area).

© 2023 Wang CJ et al. *JAMA Network Open.*

**eTable 4.** Subgroup Analysis For the Effect Of Intervention On Reperfusion Treatment For Other Secondary Outcomes.

| Outcomes | Subgroup | No.of Patients | Event in Intervention No.(%) | Event in Control No.(%) | Covariate-adjusted Anaysis[a] | | P for interaction |
|---|---|---|---|---|---|---|---|
| | | | | | Adjusted RD(95% CI) | Adjusted OR(95% CI) | |
| DNT within 60 min | Overall | 5442 | 2018 (71.3) | 1701 (65.1) | 7.1 (-5.3 to 19.4) | 1.39 (0.77–2.54) | |
| | Age,y | | | | | | 0.41 |
| | <65 | 2332 | 852 (72.1) | 747 (65.0) | 6.3 (-6.2 to 18.7) | 1.35 (0.74–2.44) | |
| | ≥65 | 3110 | 1166 (70.8) | 954 (65.3) | 7.9 (-5.0 to 20.9) | 1.44 (0.76–2.73) | |
| | Sex | | | | | | 0.21 |
| | Female | 1946 | 719 (71.7) | 600 (63.6) | 9.9 (-3.1 to 22.8) | 1.56 (0.81–3.00) | |
| | Male | 3496 | 1299 (71.1) | 1101 (66.0) | 5.5 (-7.2 to 18.1) | 1.31 (0.72–2.38) | |
| | Hospital level | | | | | | 0.31 |
| | Secondary | 1978 | 798 (75.1) | 589 (64.3) | 11.7 (-2.7 to 26.0) | 1.77 (0.73–4.27) | |
| | Tertiary | 3464 | 1220 (69.0) | 1112 (65.6) | 4.8 (-11.8 to 21.4) | 1.22 (0.57–2.58) | |
| DPT within 90 min | Overall | 506 | 62 (23.0) | 86 (36.4) | 7.1 (-5.3 to 19.4) | 0.27 (0.14–0.52) | |
| | Age,y | | | | | | 0.96 |
| | <65 | 209 | 28 (26.7) | 41 (39.4) | -17.3 (-38.0 to 3.5) | 0.42 (0.16–1.06) | |
| | ≥65 | 297 | 34 (20.6) | 45 (34.1) | -29.4 (-46.4 to -12.3) | 0.16 (0.06–0.46) | |
| | Sex | | | | | | 0.42 |
| | Female | 185 | 17 (18.5) | 31 (33.3) | -26.2 (-53.6 to 1.1) | 0.21 (0.06–0.71) | |
| | Male | 321 | 45 (25.3) | 55 (38.5) | -22.9 (-38.1 to -7.8) | 0.28 (0.12–0.66) | |
| | Hospital level | | | | | | 0.68 |
| | Secondary | 88 | 2 (3.9) | 3 (8.1) | 4.1 (-1.2 to 9.3) | NA | |
| | Tertiary | 418 | 60 (27.4) | 83 (41.7) | -25.7 (-39.3 to -12.2) | 0.28 (0.15–0.55) | |
| In-hospital death | Overall | 12 132 | 146 (2.6) | 165 (2.6) | -0.3 (-1.2 to 0.6) | 0.93 (0.61–1.41) | |
| | Age,y | | | | | | 0.08 |
| | <65 | 5186 | 20 (0.8) | 35 (1.2) | -0.8 (-1.5 to -0.1) | 0.39 (0.18–0.83) | |
| | ≥65 | 6946 | 126 (3.8) | 130 (3.6) | 0.1 (-1.2 to 1.5) | 1.08 (0.70–1.67) | |
| | Sex | | | | | | 0.90 |

© 2023 Wang CJ et al. *JAMA Network Open.*

| | | | | | | | |
|---|---|---|---|---|---|---|---|
| | Female | 4373 | 63 (3.1) | 73 (3.1) | 0.4 (-1.0 to 1.7) | 1.02 (0.60–1.75) | |
| | Male | 7759 | 83 (2.3) | 92 (2.2) | -0.6 (-1.5 to 0.3) | 0.89 (0.54–1.45) | |
| | Hospital level | | | | | | 0.72 |
| | Secondary | 4072 | 46 (2.5) | 53 (2.4) | -0.1 (-1.3 to 1.2) | 1.22 (0.67–2.23) | |
| | Tertiary | 8060 | 100 (2.6) | 112 (2.6) | -0.5 (-1.6 to 0.6) | 0.84 (0.48–1.47) | |
| Disability at 90d | Overall | 10 398 | 1215 (25.9) | 1439 (25.2) | -0.9 (-5.1 to 3.2) | 0.96 (0.77–1.20) | |
| | Age,y | | | | | | 0.04 |
| | <65 | 4576 | 299 (14.8) | 415 (16.2) | -2.8 (-5.7 to 0.2) | 0.81 (0.63–1.02) | |
| | ≥65 | 5822 | 916 (34.4) | 1024 (32.4) | 0.2 (-4.6 to 5.0) | 1.02 (0.82–1.26) | |
| | Sex | | | | | | 0.71 |
| | Female | 3744 | 532 (31.5) | 623 (30.3) | 0.8 (-4.2 to 5.7) | 1.04 (0.82–1.32) | |
| | Male | 6654 | 683 (22.8) | 816 (22.3) | -1.8 (-5.9 to 2.4) | 0.92 (0.72–1.17) | |
| | Hospital level | | | | | | 0.10 |
| | Secondary | 3484 | 448 (28.7) | 490 (25.5) | 3.0 (-4.1 to 10.1) | 1.18 (0.82–1.70) | |
| | Tertiary | 6914 | 767 (24.5) | 949 (25.1) | -2.9 (-8.2 to 2.3) | 0.87 (0.66–1.16) | |

Abbreviations: DNT: door-to-needle time; DPT: door-to-punctuation time.

[a] Results were yielded from generalized estimating equation logistic regression models with adjustment for the hospital clustering effect, temporal period, and imbalanced baseline covariates (medical history of stroke or transient ischemic attack, medication history of antiplatelet agent and statin, and hospital area).

© 2023 Wang CJ et al. *JAMA Network Open.*

**eTable 5.** Baseline Characteristics of the IMPROVE Stroke Care in China Patients and Hospitals by Reperfusion.

| Characteristics | No IV/EV (N=6256 [51.6%]) | IV/EV (N=5876 [48.4%]) | Differece (95% CI) |
|---|---|---|---|
| **Patient characteristics** | | | |
| Demographic | | | |
| Age, mean (SD), y | 66.1±12.2 | 66.1±11.9 | 0.0 (-0.4 to 0.5) |
| Male | 3985 (63.7) | 3774 (64.2) | -0.5 (-2.2 to 1.2) |
| BMI, mean (SD), kg/m2 | 24.1±4.0 | 23.4±3.6 | 0.7 (0.5 to 0.8) |
| NIHSS at admission | 3.0 (2.0–6.0) | 6.0 (3.0–12.0) | -3.0 (-3.0 to -2.0) |
| Current smoking | 1561 (25.0) | 1677 (28.5) | -3.6 (-5.2 to -2.0) |
| Drinking | 1561 (25.0) | 1432 (24.4) | 0.6 (-1.0 to 2.1) |
| Medical history | | | |
| Stroke or TIA | 2207 (35.3) | 1238 (21.1) | 14.2 (12.6 to 15.8) |
| Hypertension | 3908 (62.5) | 3549 (60.4) | 2.1 (0.3 to 3.8) |
| Diabetes | 1381 (22.1) | 1020 (17.4) | 4.7 (3.3 to 6.1) |
| Dyslipidemia | 408 (6.5) | 317 (5.4) | 1.1 (0.3 to 2.0) |
| Myocardial infarction | 126 (2.0) | 116 (2.0) | 0.0 (-0.5 to 0.5) |
| Atrial fibrillation | 441 (7.0) | 666 (11.3) | -4.3 (-5.3 to -3.3) |
| Peripheral vascular disease | 124 (2.0) | 41 (0.7) | 1.3 (0.9 to 1.7) |
| Medication history | | | |
| Antiplatelet agent | 1492 (23.8) | 758 (12.9) | 10.9 (9.6 to 12.3) |
| Anticoagulant drugs | 399 (6.4) | 153 (2.6) | 3.8 (3.0 to 4.5) |
| Antihypertensive drugs | 2865 (45.8) | 2512 (42.8) | 3.0 (1.3 to 4.8) |
| Antidiabetics | 1110 (17.7) | 794 (13.5) | 4.2 (2.9 to 5.5) |
| Statins | 1348 (21.5) | 613 (10.4) | 11.1 (9.8 to 12.4) |
| Onset to door time,min | | | |
| **Hospital characteristics** | | | |
| Hospital level | | | |
| Tertiary | 4210 (67.3) | 3850 (65.5) | 1.8 (0.1 to 3.5) |
| Secondary | 2046 (32.7) | 2026 (34.5) | -1.8 (-3.5 to -0.1) |
| Hospital location | | | |
| Eastern | 2910 (46.5) | 2664 (45.3) | 1.2 (-0.6 to 3.0) |
| Central | 1981 (31.7) | 1683 (28.6) | 3.0 (1.4 to 4.7) |
| Western | 1365 (21.8) | 1529 (26.0) | -4.2 (-5.7 to -2.7) |

Abbreviation: SD, standard deviation; BMI: body mass index; NIHSS, the National Institutes of Health Stroke Scale. TIA, transient ischemic attack.

© 2023 Wang CJ et al. *JAMA Network Open.*

**eTable 6.** Results of Disability at 90 Days From Raw Data.

| Outcomes | Reperfusion | | Raw Analysis | | Primary Analysis[a] | | Covariates-Adjusted Analysis[b] | |
|---|---|---|---|---|---|---|---|---|
| | Yes | No | RD (95% CI) | OR (95% CI) | RD (95% CI) | OR (95% CI) | RD (95% CI) | OR (95% CI) |
| Disability at 90d | 1381/4980 (27.7) | 1273/5418 (23.5) | 4.2 (1.6 to 6.8) | 1.25 (1.09–1.44) | 4.1 (1.6 to 6.6) | 1.24 (1.08-1.42) | -4.0 (-6.1 to -1.9) | 0.80 (0.70–0.91) |

[a] Results were yielded from generalized estimating equation logistic regression models with adjustment for the hospital clustering effect and temporal period.

[b] Results were yielded from generalized estimating equation logistic regression models with adjustment for the hospital clustering effect, temporal period, and imbalanced baseline covariates (medical history of stroke or transient ischemic attack, medication history of antiplatelet agent and statin, and hospital area).

© 2023 Wang CJ et al. *JAMA Network Open.*

| Potential causes for the low rate of reperfusion therapy in China | Designed STEP interventions component |
|---|---|
| * The unorganized stroke care in stroke centers | ➢ **S**trategies: a group of best practice strategies developed from the "Target: Stroke Initiative"; an official written documents provided to receive administrative support from the head of public hospitals |
| * The eroded patient-physician trust that encountered during structural and social transformations | ➢ **T**oolkit: an integrated toolkit presented as a handbook and designed according to the American Heart Association/American Stroke Association and Chinese Stroke Association guidelines, including an informed consent form with full consideration of medical humanities for intravenous thrombolysis/endovascular thrombectomy . |
| * Unequal distribution of high-quality health resources | ➢ **E**xploration: an explorative intervention strategy of providing point-to-point experienced expert guidance to the hospitals entered into the intervention stage |
| * The unorganized stroke care in stroke centers | ➢ **P**aradigm: the continuously improved paradigm of the feedback system served to make a Plan-Do-Study-Act circle |

**eFigure 1.** The Design Of "STEP" Intervention.
STEP indicates Strategies, Toolkit, Exploration, and Paradigm.

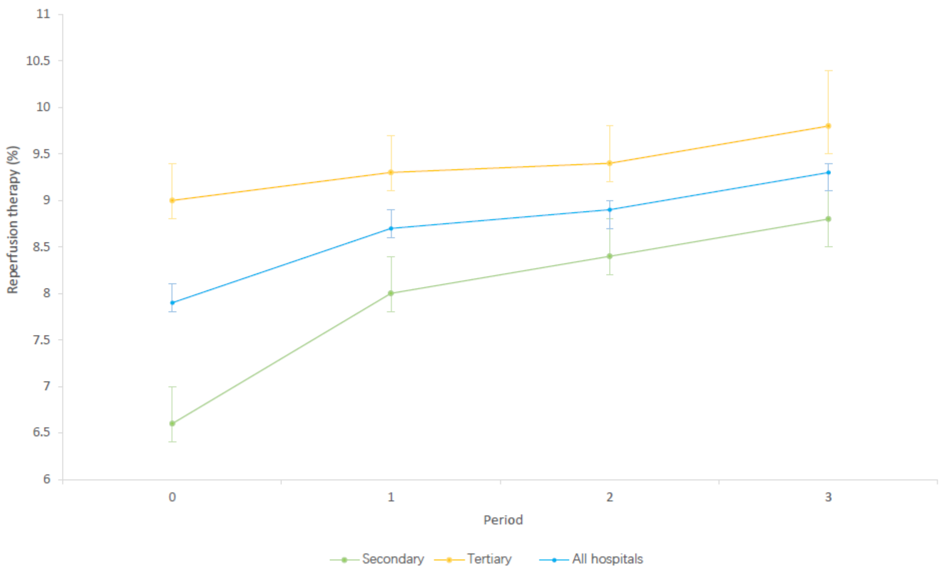


**eFigue 2.** Reperfusion Therapy In The Chinese Center Association Alliance Hospitals Not Participating in the IMPROVE Stroke Care In China Trial

© 2023 Wang CJ et al. *JAMA Network Open.*

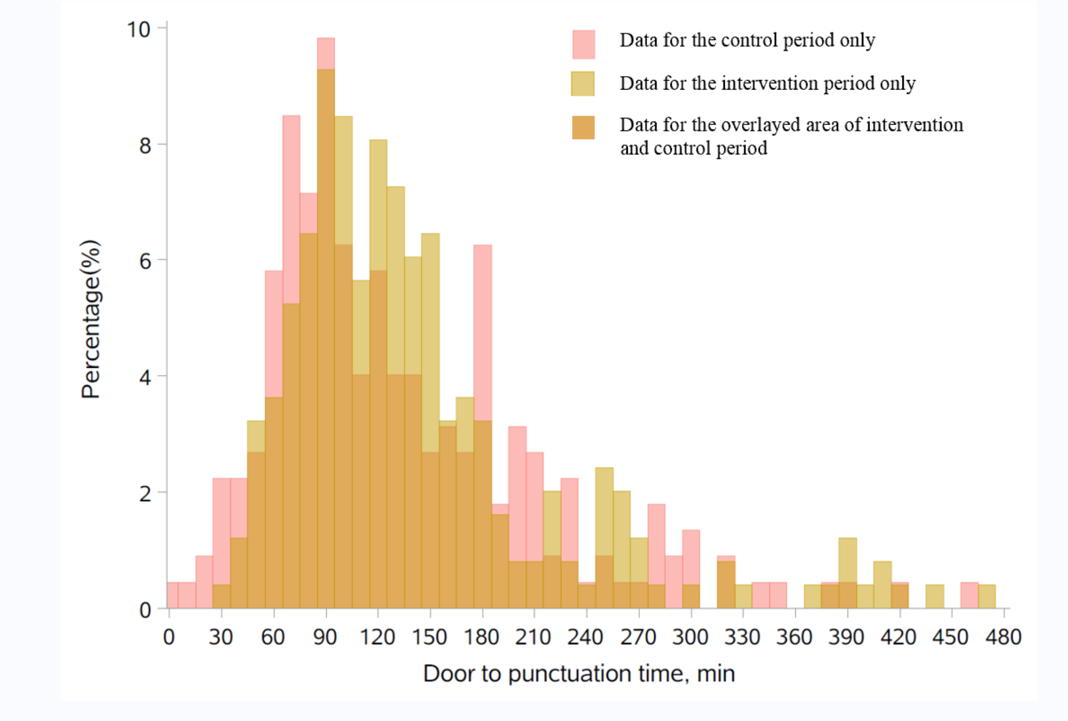


**eFigue 3.** Distribution of Door-to-Puncture Time in the IMPROVE Stroke Care in China Trial.

© 2023 Wang CJ et al. *JAMA Network Open.*
