# Supplementary material for: Effectiveness of a Quality Improvement Intervention on Reperfusion Treatment for Patients With Acute Ischemic Stroke: A Stepped-Wedge Cluster Randomized Clinical Trial
Source: JAMA Netw Open. 2023 Jun 2;6(6):e2316465. doi: 10.1001/jamanetworkopen.2023.16465 (PMC10238948; doi:10.1001/jamanetworkopen.2023.16465)
Supplement: Supplement 3. — Nonauthor Collaborators [file jamanetwopen-e2316465-s003.pdf]

Supplemental Online Content: Nonauthor Collaborators
*First name, last name, and suffix (if applicable) are required and will appear in PubMed.

*Group Name(s): IMPROVE Stroke Care in China investigators							
***First Name and Middle Initial(s)**	***Last Name**	***Suffix (eg, Jr, III)**	Academic Degrees	Institution	Location (city, state/province, country)	Role or Contribution, eg, chair, principal investigator	Group (if more than 1 Group listed in the byline) and/or Subgroup (eg, Steering Committee)
Qingcheng	Yang		MD	People's Hospital of Anyang city	Anyang, Henan, China	Site Investigator	
Guangsheng	Chen		MD	People's Hospital fo boluo County	Boluo County, Guangdong, China	Site Investigator	
Qianli	Ma		MD	People's Hospital of haining city	Haining, Zhejiang, China	Site Investigator	
Xiaobo	Li		MD	Subei People's Hospital of Jiangsu province	Yangzhou, Jiangsu, China	Site Investigator	
Jun	Chen		MD	The First Hospital of Lanzhou University	Lanzhou, Gansu, China	Site Investigator	
Xiuli	Zhao		MD	Affiliated Hospital of Qinghai University	Xining,Qinghai, China	Site Investigator	
Hongping	Wang		MD	The First People's Hospital of Qingzhen city	Qingzhen, Guizhou, China	Site Investigator	
Xiaoyuan	Niu		MD	First Hospital of Shanxi Medical University	Taiyuan,Shanxi, China	Site Investigator	
Jianhua	Xu		MD	Jiading District Central Hospital of Shanghai city	Shanghai, China	Site Investigator	
Lile	Zhao		MD	Xiqing hospital of Tianjin city	Tianjin, China	Site Investigator	
Zicheng	Wang		MD	People's Hospital of Wuzhong city	Wuzhong, Ningxia, China	Site Investigator	
Danqin	Huang		MD	Hospital of Wuyishan City	Wuyishan, Fujian, China	Site Investigator	
Xiaoping	Jin		MD	Taizhou Hospital of Zhejiang Province	Taizhou, Zhejiang, China	Site Investigator	
Shengli	Chen		MD	Three Gorges Center Hospital of Chongqing	Chongqing, China	Site Investigator	
Jianhua	Li		MD, PhD	The First Hospital of Fangshan District	Beijing, China	Site Investigator	
Juming	Yu		MD	Affiliated hospital of north sichuan medical college	Nanchong, Sichuan, China	Site Investigator	

Supplemental Online Content: Nonauthor Collaborators
*First name, last name, and suffix (if applicable) are required and will appear in PubMed.

***First Name and Middle Initial(s)**	***Last Name**	***Suffix (eg, Jr, III)**	Academic Degrees	Institution	Location (city, state/province, country)	Role or Contribution, eg, chair, principal investigator	Group (if more than 1 Group listed in the byline) and/or Subgroup (eg, Steering Committee)
Ping	Liu		MD	Dali bai autonomous prefecture people's hospital	Dali, Yunnan, China	Site Investigator	
Guozhong	Li		MD	The First Clinical Hospital affiliated to Harbin Medical University	Harbin, Heilongjiang, China	Site Investigator	
Yanlei	Hao		MD	Affiliated Hospital of Jining Medical University	Jining, Shandong, China	Site Investigator	
Guancheng	Yang		MD	People's Hospitalof jishan County	Jishan county, Shanxi, China	Site Investigator	
Xiaoxin	Huang		MD	Jieyang city People's Hospital	Jieyang, Guangdong, China	Site Investigator	
Chengfang	Zhou		MD	The second hospital, university of south China	Changsha, Hunan, China	Site Investigator	
Junsu	Yang		MD	The First People's Hospital of Qujing city	Qujing, Yunnan, China	Site Investigator	
Jun	Gu		MD	Jiangsu rudong county People's Hospital	Rudong, Jiangsu, China	Site Investigator	
Peng	Sun		MD	Sanya people's hospital	Sanya, Hainan, China	Site Investigator	
Zaiyu	Guo		MD	Tianjin Teda hospital	Tianjin, China	Site Investigator	
Guoping	Ma		MD	First People's Hospital of Tianshui city	Tianshui, Gansu, China	Site Investigator	
Guohua	Chen		MD	Wuhan No. 1 Hospital	Wuhan, Hubei, China	Site Investigator	
Mingshan	Tang		MD	Banan People's Hospital of Chongqing	Chongqing, China	Site Investigator	
Ning	Wang		MD	The first affiliated hospital of fujian medical university	Fuzhou, Fujian, China	Site Investigator	
Lixia	Chen		MD	The 2nd Affiliated Hospital of Harbin Medical University	Harbin, Heilongjiang, China	Site Investigator	
Juntao	Li		MD	Handan Central Hospital	Handan, Hebei, China	Site Investigator	
Aiwu	Li		MD	People's Hospital of zunhua	Zunhua, Hebei, China	Site Investigator	
Song	Li		MD	Jilin Province People's Hospital	Changchun, Jilin, China	Site Investigator	
Minghua	Cao		MD	The First People's Hospital of Jingdezhen city	Jingdezhen, Jiangxi, China	Site Investigator	

Supplemental Online Content: Nonauthor Collaborators
*First name, last name, and suffix (if applicable) are required and will appear in PubMed.

***First Name and Middle Initial(s)**	***Last Name**	***Suffix (eg, Jr, III)**	Academic Degrees	Institution	Location (city, state/province, country)	Role or Contribution, eg, chair, principal investigator	Group (if more than 1 Group listed in the byline) and/or Subgroup (eg, Steering Committee)
Jianqiang	Guo		MD	The Fourth People's Hospital of Langfang city	Langfang, Hebei, China	Site Investigator	
Youquan	Ren		MD	People's Hospital of Linquan County	Linquan County, Anhui, China	Site Investigator	
Tong	Li		MD	The second nanning People's Hospita	Nanning, Guangxi, China	Site Investigator	
Lihong	Zhang		MD	Shenyang 739 Hospital	Shenyang, Liaoning, China	Site Investigator	
Zhaoming	Xie		MD	People's Hospital of Tengchong city	Tengchong, Yunnan, China	Site Investigator	
Junlin	Dong		MD	People's Hospital of Wusu city	Wusu, Xinjiang, China	Site Investigator	
Xiangfeng	Kong		MD	Xinjiang Medical University Fifth Affiliated Hospital	Urumqi, Xinjiang, China	Site Investigator	
Hui	Liang		MD	Yantai Mountain Hospital	Yantai, Shandong, China	Site Investigator	
Yan	Zhang		MD	People's Hospital of Qinan County	Qinan County, Shandong, China	Site Investigator	
