# Supplementary material for: Effectiveness of a Quality Improvement Intervention on Reperfusion Treatment for Patients With Acute Ischemic Stroke: A Stepped-Wedge Cluster Randomized Clinical Trial
Source: JAMA Netw Open. 2023 Jun 2;6(6):e2316465. doi: 10.1001/jamanetworkopen.2023.16465 (PMC10238948; doi:10.1001/jamanetworkopen.2023.16465)
Supplement: Supplement 4. — Data Sharing Statement [file jamanetwopen-e2316465-s004.pdf]

Data Sharing Statement

Wang. Effectiveness of a Quality Improvement Intervention on Reperfusion Treatment for Patients With Acute Ischemic Stroke. *JAMA Netw Open*. Published June 02, 2023. doi:10.1001/jamanetworkopen.2023.16465

Data
Data available: Yes
Data types: Deidentified participant data, Data dictionary
How to access data: yongjunwang@ncrcnd.org.cn
When available: With publication

Supporting Documents
Document types: None

Additional Information
Who can access the data: yongjunwang@ncrcnd.org.cn
Types of analyses: For the purpose of repeating the current analysis
Mechanisms of data availability: with a signed data access agreement
Any additional restrictions: For the purpose of repeating the current analysis
